# *In silico* virtual screening for the identification of novel inhibitors against dihydrodipicolinate reductase (DapB) of *Mycobacterium tuberculosis*, a key enzyme of diaminopimelate pathway

**DOI:** 10.1128/spectrum.01359-23

**Published:** 2023-10-19

**Authors:** Nupur Angrish, Neha Lalwani, Garima Khare

**Affiliations:** 1 Department of Biochemistry, University of Delhi South Campus, New Delhi, India; Johns Hopkins University School of Medicine, Baltimore, Maryland, USA

**Keywords:** tuberculosis, *Mycobacterium tuberculosis*, virtual screening

## Abstract

Tuberculosis (TB) caused by *Mycobacterium tuberculosis* (*M. tb*) is one of the most devastating infectious diseases afflicting a large number of human lives. In spite of the availability of several drugs for treating TB, emergence of drug-resistant strains of the pathogen has made treatment and eradication of TB a challenging task. Hence, there is an imperative need for new intervention strategies to target *M. tb*. The enzymes of the diaminopimelate pathway, involved in lysine biosynthesis as well as in cell wall biosynthesis, have been considered as important anti-TB drug targets. One such enzyme is dihydrodipicolinate reductase (DapB), catalyzing the reduction of 2,3-dihydrodipicolinic acid to 2,3,4,5-tetrahydro-dipicolinic acid by utilizing NADH/NADPH as its cofactor. Here, we have generated an antisense knockdown mutant strain of *dapB* and demonstrated a crucial role of *dapB* in the *in vitro* and intracellular growth of *M. tb*. Further, *in silico* virtual screening was performed by employing a library of ~95,000 compounds for the identification of inhibitory molecules against DapB. The molecules with high docking scores were screened against the enzymatic activity of DapB to determine their IC_50_ values. Further, hit molecules that inhibited DapB were screened against the *M. tb* growth in broth culture and inside macrophages. The molecules exhibiting *M. tb* inhibition were also evaluated for their cytotoxicity against various mammalian cell lines. In summary, we have identified a lead molecule, B59, with an IC_50_ value of 11 µg/mL and MIC_99_ value of 20 µg/mL, which can be further optimized to develop potent inhibitory compounds against *M. tb* DapB.

**IMPORTANCE:**

Non-compliance to lengthy antituberculosis (TB) treatment regimen, associated side effects, and emergence of drug-resistant strains of *Mycobacterium tuberculosis* (*M. tb*) emphasize the need to develop more effective anti-TB drugs. Here, we have evaluated the role of *M. tb* dihydrodipicolinate reductase (DapB), a component of the diaminopimelate pathway, which is involved in the biosynthesis of both lysine and mycobacterial cell wall. We showed that DapB is essential for the *in vitro* as well as intracellular growth of *M. tb*. We further utilized *M. tb* DapB, as a target for identification of inhibitors by employing *in silico* virtual screening, and conducted various *in vitro* screening assays to identify inhibitors with potential to inhibit DapB activity and *in vitro* and intracellular growth of *M. tb* with no significant cytotoxicity against various mammalian cell lines. Altogether, *M. tb* DapB serves as an important drug target and a hit molecule, namely, 4-(3-Phenylazoquinoxalin-2-yl) butanoic acid methyl ester has been identified as an antimycobacterial molecule in our study.

## INTRODUCTION

In spite of the enormous efforts during the last several decades, *Mycobacterium tuberculosis* (*M. tb*) still caused 1.51 million human deaths due to tuberculosis (TB) globally in the year 2021 (1). Additionally, the rise of the COVID-19 pandemic has negatively impacted the overall progress of TB control that led to a rise in the TB mortality rate reversing the gains achieved in the last 5 years (1). The 6–9 months lengthy anti-TB chemotherapy leads to non-compliance to the therapy resulting in the emergence of multidrug-resistant (MDR) and extremely drug-resistant (XDR) strains of *M. tb* (2). Hence, the situation demands to strengthen the anti-TB drug discovery efforts for the search of more efficacious anti-TB molecules for an effective control of TB.

The major challenge for drug discovery often lies in the identification of an appropriate target that is essential for the pathogen’s survival or is involved in the virulence-related pathways of mycobacteria. Among several metabolic pathways, genes involved in essential amino acid biosynthesis are suitable for targeting pathogen as these amino acids are essential for the survival and pathogenesis of *M. tb* and have no human homolog (3). Apart from this, enzymes that are involved in cell wall biosynthesis of mycobacteria are considered as attractive drug targets as components of cell wall are the major virulence factors acting as a physical barrier for drugs or antimicrobial agents (4, 5). One of the important pathways in mycobacteria is the diaminopimelate (DAP) pathway that is involved in both the biosynthesis of meso-diaminopimelate (meso-DAP) as well as essential amino acid, lysine. Meso-diaminopimelate serves as an important crosslink component in the peptidoglycan layer of mycobacteria. Moreover, this pathway is absent in the mammalian system, hence, targeting enzymes involved in the DAP pathway would serve as an important stride toward anti-mycobacterial drug discovery research (6, 7).

**Fig 1:**
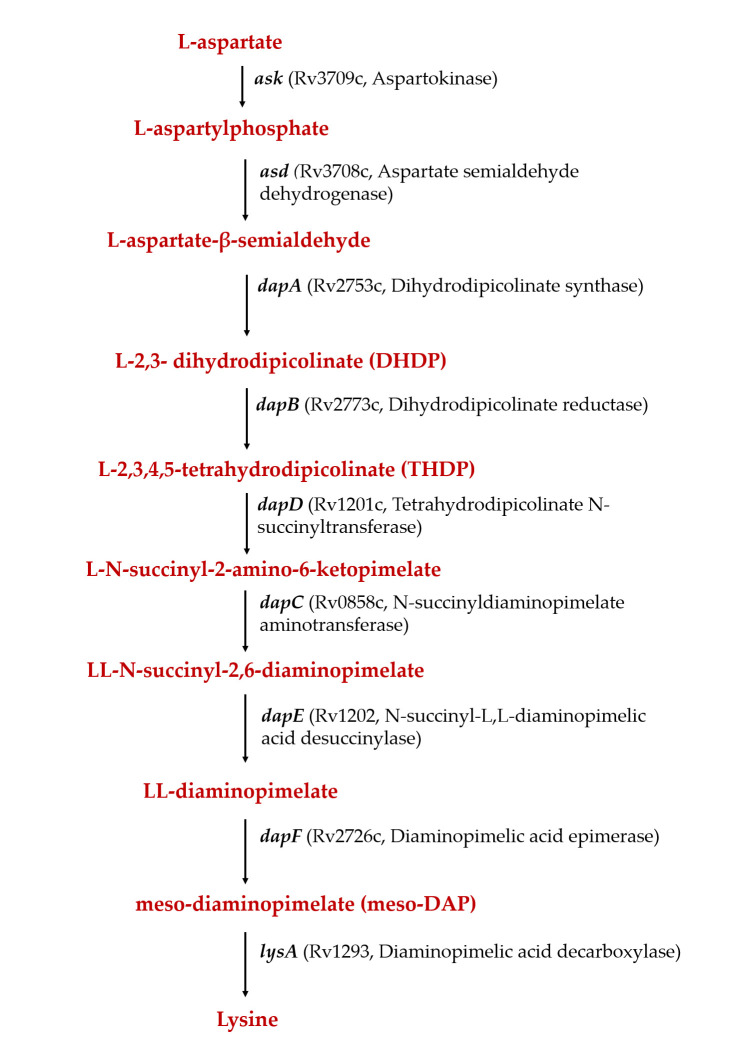
Schematic diagram showing various steps of the diaminopimelate pathway in *Mycobacterium tuberculosis*.

The pathway involves nine enzymatic steps and begins with the phosphorylation of L-aspartate by aspartokinase (Ask) with subsequent reduction of L-β-aspartyl phosphate to L-aspartate-β-semialdehyde (ASA) catalyzed by aspartate semialdehyde dehydrogenase (Asd) (Fig. 1). This is followed by aldol condensation reaction between pyruvate and ASA to form L-2,3-dihydrodipicolinate (DHDP) catalyzed by dihydrodipicolinate synthase (DapA). Subsequently, DHDP is reduced to L-2,3,4,5-tetrahydrodipicolinate (THDP) in a reaction catalyzed by dihydrodipicolinate reductase (DapB) by using pyridine nucleotide as an electron donor (Fig. S1) (8). L-2,3,4,5-Tetrahydrodipicolinate is further converted to meso-DAP either through a single reaction (dehydrogenase pathway) or through multiple steps (succinylase pathway and acetylase pathway); the latter is further utilized for the formation of either lysine or peptidoglycan (9).

Several transposon mutagenesis studies showed that the genes involved in the DAP pathway are essential for the growth of mycobacteria (10 – 12). It was also demonstrated by Pavelka et al. that disruption of the first gene (*ask*) of this pathway in *Mycobacterium smegmatis* led to several auxotrophies (Met^-^, Thr^-^, DAP^-^, and Lys^-^), suggesting the essentiality of this pathway in mycobacteria (13). Hence, meso-DAP is suggested as an important metabolic intermediate that is central to peptidoglycan synthesis and thus appears to be essential for the pathogen’s survival. In this study, we made an attempt to understand the importance of the DAP biosynthesis pathway in *M. tb* by specifically studying the role of DapB, which is required for the synthesis of THDP, the precursor molecule for meso-DAP.

*dapB* (*rv2773c*) encodes dihydrodipicolinate reductase, which catalyzes the reduction of α,β-unsaturated cyclic imine 2,3-dihydrodipicolinic acid to 2,3,4,5-tetrahydrodipicolinic acid. The crystal structures of DapB for various organisms including *Escherichia coli*, *Thermotoga maritima*, *Corynebacterium glutamicum*, *Neisseria gonorrhoeae*, *Staphylococcus aureus*, *Acinetobacter baumannii*, and *M. tuberculosis* are available in the Protein Data Bank (PDB) database (14 – 19). The three-dimensional structure of *M. tb* DapB was determined by using molecular replacement strategy as a ternary complex with NADH-2,6-PDC (PDB ID: 1P9L) and with NADPH-2,6-PDC (PDB ID:1C3V) in a closed conformation (18). The overall structure consists of four identical subunits, and each subunit comprises of two domains that are connected through two flexible hinge regions. The N-terminal domain is responsible for pyridine nucleotide binding and is composed of amino acid residues, Met1-Ala106 and Ser216-His245. The C- terminal region consists of 109 amino acids (Ile107-Thr-215) which are involved in tetramerization as well as in substrate/inhibitor binding. It was observed that mycobacterial DapB can use both NADH and NADPH with nearly equal efficiency with *K*_m_ values of 3.2 ± 0.4 µM and 11.8 ± 1.5 µM, respectively (18). Later, the structure was also determined in its apo form and as a binary complex with NADH (19). It was observed that the apoenzyme mainly exists in the open conformation and the presence or absence of cofactor/substrate/inhibitor decides the conformation of the enzyme. Further, Paiva et al. have identified inhibitors of *M. tb* DapB by employing a combination of the molecular modeling approach and conventional screening of a library, resulting in the identification of several sulphonamide molecules with Ki values ranging from 7 to 48 µM (20). However, these compounds were not evaluated for their ability to inhibit the growth of *M. tb*. Recently, a study compared conventional pharmacophore models with the dynamic hybrid pharmacophore model to screen a library of 15,63,764 molecules against DapB (21). It was noted that hybrid dynamic-based pharmacophore strategy yielded molecules with new chemotypes and better binding affinities and having drug-like properties. However, these molecules were not evaluated against the enzymatic activity of DapB (21).

In the present study, we investigated the role of *dapB* in the *in vitro* as well as intracellular growth of *M. tb* by employing a *dapB* antisense knockdown mutant strain of *M. tb*. Our data for the first time directly demonstrate the importance of *dapB* in the growth of mycobacteria as *dapB* knockdown mutant strain exhibited attenuated growth *in vitro* as well as inside macrophages. Further, we attempted to identify inhibitory molecules against DapB by employing an *in silico* target-based virtual screening approach. The top-ranking molecules from the docking studies were assessed for their inhibitory potential against the activity of DapB by employing an optimized biochemical enzymatic assay. Moreover, the lead molecules obtained were evaluated against the growth of *M. tb in vitro* and inside THP-1 macrophages followed by evaluation of their cytotoxicity against various mammalian cell lines. Taken together, our work identified a hit molecule against *M. tb* DapB which can be further optimized by performing structure activity relationship studies for designing a potent inhibitor against *M. tb* DapB.

## RESULTS

### Generation and characterization of *M. tb dapB* antisense knockdown mutant strain

**Fig 2:**
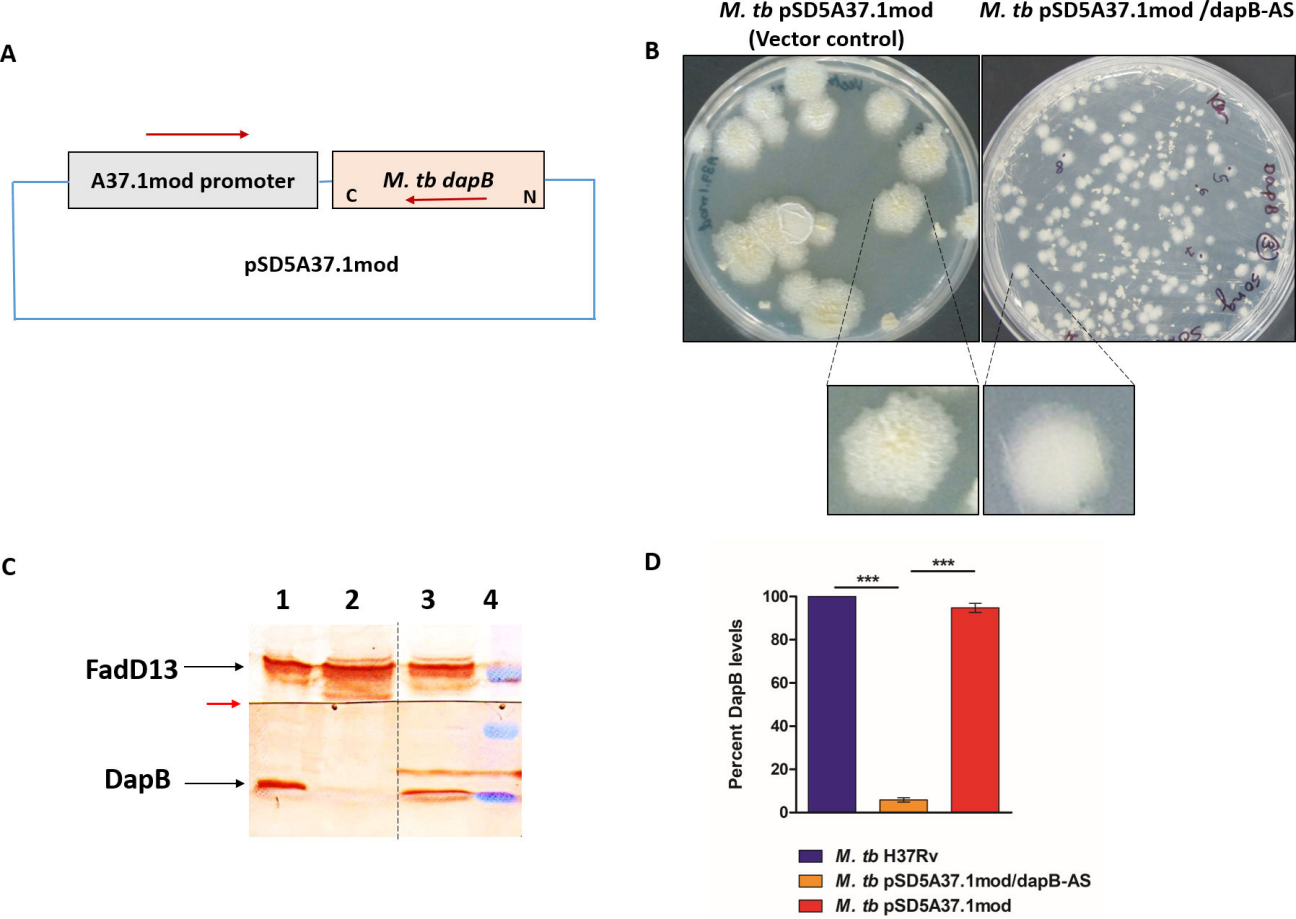
Construction of *M. tb dapB* knockdown mutant strain. (**A**) Schematic representation showing dapB gene cloned in an antisense orientation downstream to an A37.1mod promoter in a pSD5A37.1mod promoter. (**B**) Colony morphology of *M. tb* vector control strain (*M. tb* pSD5A37.1mod) and antisense knockdown mutant strain (*M. tb* pSD5A37.1mod/dapB-AS). dapB antisense mutant strain showed smooth and small-sized colonies as compared with rough and large-sized colonies in vector control. (**C**) Confirmation of *dapB* antisense knockdown in *M. tb* by immunoblot analysis. Lysates containing 70 µg protein each of *M. tb* (lane 1), *M. tb* pSD5A37.1mod/dapB-AS (lane 2), and *M. tb* pSD5A37.1mod (lane 3) were electrophoresed on 12.5% polyacrylamide gel. The proteins were transferred on PVDF (polyvinylidene difluoride) membrane, and the blot was cut into two at the red arrow. Immunoblotting was carried out by using anti-DapB antibody for the lower part of the membrane and with anti-fadD13 antibody for the upper part. *Mycobacterium tuberculosis* FadD13, a mycobacterial cytosolic protein unrelated to the DAP pathway, was employed as a loading control. Dashed line shows that different lanes (non-adjacent) from the same immunoblot have been brought together. Lane 4—pre-stained protein molecular weight markers (from the top: 50, 37, and 25 kDa). (**D**) The bar diagram shows percent inhibition of DapB protein levels in *M. tb* vector control and antisense knockdown mutant strain as compared with *M. tb* H37Rv after normalizing the data with FadD13 levels (loading control). Quantification of the protein levels in the immunoblot was performed by using ImageJ software. The data are represented as the mean ± SEM (error bars) of two independent experiments, and representative immunoblot is shown.

In order to elucidate the role of *dapB* in *M. tb* physiology and pathogenesis, we made an attempt to generate *dapB* knockout strain of *M. tb* by homologous recombination approach. Unfortunately, we were unable to obtain knockout mutant colonies even after three separate attempts of homologous recombination highlighting a crucial role of DapB for the growth of *M. tb*. Additionally, a study by DeJesus et al. also identified a region containing *dapB* as an essential ORF (open reading frame) for *M. tb* by creating a completely saturated transposon mutants’ library in *M. tb* (12). Hence, we constructed a knockdown mutant strain of *dapB* by antisense strategy (22). For this, the coding sequence of the *dapB* gene was amplified by PCR using *M. tb* H37Rv genomic DNA by employing dap-F-Mlu1 and dap-R-Nde1 primers and cloned in pSD5A37.1mod vector resulting into a recombinant antisense plasmid carrying *dapB* in opposite orientation with respect to a strong A37.1mod promoter (Fig. 2A; Fig. S2A) (23, 24). Further, the screening of recombinant *E. coli* colonies was carried out by restriction digestion that gave a fallout of ~700 bp corresponding to the size of the insert (Fig. S2B). The recombinant plasmid pSD5A37.1mod/dapB-AS and empty vector were employed separately for electroporation of *M. tb* competent cells. It was observed that the colonies of *dapB* knockdown mutant strain displayed an altered morphology with smooth and small-sized colonies as compared with vector control colonies, which showed characteristic rough colony morphology of mycobacteria (Fig. 2B). For the validation of the mutant strain, plasmid DNA was isolated from the mutant colonies and screened by PCR amplification by employing two sets of primers; firstly, using A37.1mod promoter-specific forward and reverse primers yielded an amplified product of ~100 bp corresponding to the size of the A37.1mod promoter in vector control as well as in knockdown mutant strain (Fig. S2C). Secondly, the antisense mutant colonies were screened by utilizing A37.1mod promoter-specific forward primer and gene specific reverse primer, which gave an amplified product of 300 bp in the *M. tb* pSD5A37.1mod/dapB-AS strain as shown in Fig. S2D.

For further confirmation and evaluation of the effect of antisense construct on the DapB protein synthesis, western blot analysis was performed by using anti-DapB antibody. Bacterial cultures were grown to mid-log phase and whole-cell protein lysate was prepared as described in Materials and Methods. It was observed by immunoblot analysis that there was more than 90% reduction in DapB protein levels (~32 kDa) in the antisense knockdown mutant strain (*M. tb* pSD5A37.1mod/dapB-AS) as compared with *M. tb* H37Rv (Fig. 2C and D). Additionally, as expected, there was no significant difference in the DapB levels in lysates of *M. tb* H37Rv and *M. tb* H37Rv vector control (Fig. 2C).

### *Mycobacterium tuberculosis dapB* knockdown mutant strain displayed growth defect *in vitro* and inside macrophages

**Fig 3:**
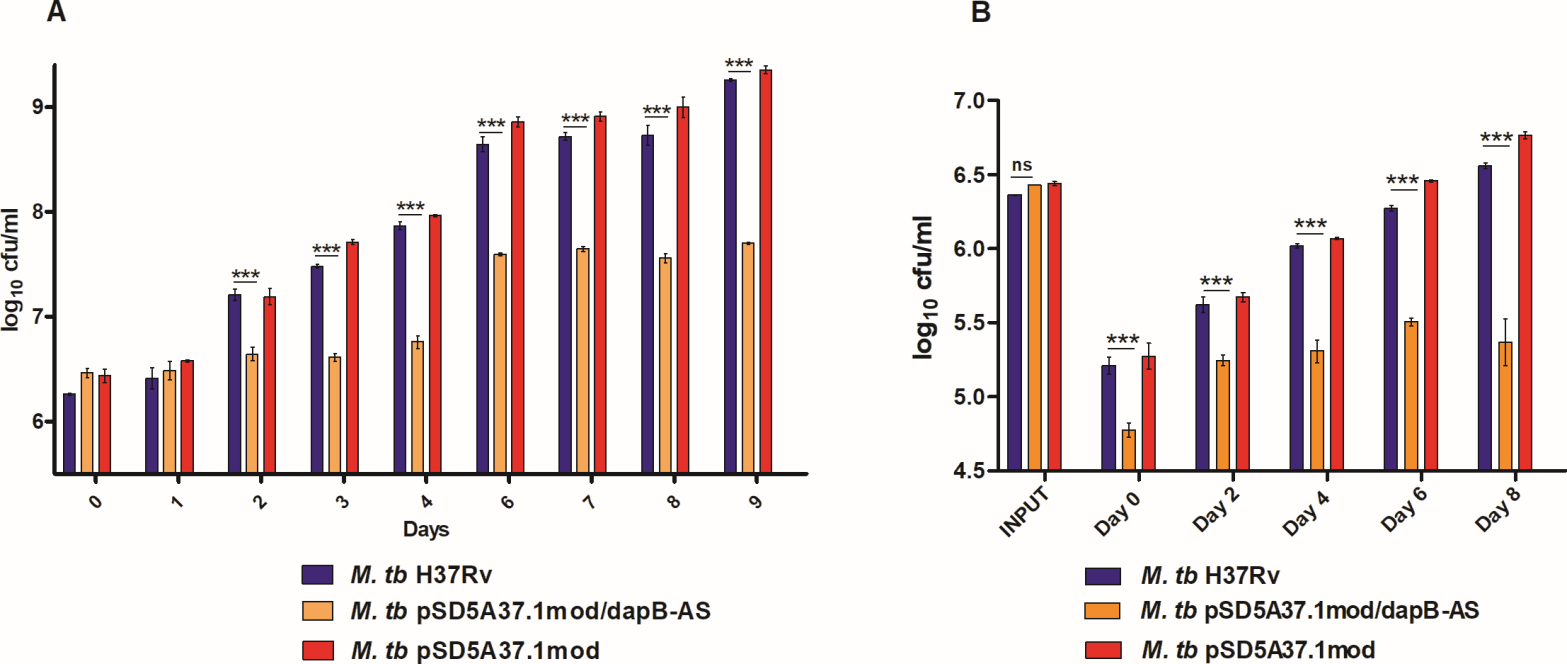
Involvement of DapB in *in vitro* and intracellular growth of *M. tb*. (**A**) *In vitro* growth of *M. tb* H37Rv (blue), *M. tb* pSD5A37.1mod/dapB-AS (orange), and *M. tb* pSD5A37.1mod (red) strains in 7H9 medium at 37°C, 200 rpm by CFU analysis. Graph showed significant difference in the growth of antisense knockdown mutant strain as compared with the wild-type *M. tb* and vector control strains. (**B**) Intracellular growth of *M. tb* H37Rv (blue), *M. tb* pSD5A37.1mod/dapB-AS (orange), and *M. tb* pSD5A37.1mod (red) strains inside THP-1 macrophages at different time points. A significant difference in the intracellular growth of *M. tb* H37Rv (blue) and *M. tb* pSD5A37.1mod/dapB-AS (orange) strains was observed. The data are represented as the mean ± SEM (error bars) of at least two independent experiments. (*P < 0.05, **P < 0.01, and ***P < 0.001, two-way ANOVA, Bonferroni post-tests).

DapB is involved in lysine biosynthesis and plays a key role in *M. tb* cell wall biosynthesis; thus, we expected that a knockdown of *dapB* might have an influence on the growth of mycobacteria. Hence, we performed a growth kinetic study for *M. tb* H37Rv, *M. tb* pSD5A37.1mod/dapB-AS, and vector control strains in MB7H9 medium under standard growth conditions. It was observed that *dapB* knockdown mutant strain demonstrated a growth defect as compared with the parental strain and the vector control strains with significantly reduced CFU values than the control strains (Fig. 3A).

Further, we also evaluated the growth kinetics of all the strains inside macrophages, which are considered as the first line of immune defense cells against *M. tb* infection (25). For this, THP-1 macrophages were infected separately with *M. tb* H37Rv, *M. tb* pSD5A37.1mod/dapB-AS, and vector control strains at an multiplicity of infection (MOI) of 1:5 and the intracellular growth of the bacteria was monitored at various time points (days 0, 2, 4, 6, and 8). It was observed that the *dapB* knockdown mutant strain exhibited a significantly reduced CFU at day 0 as compared with *M. tb* H37Rv and vector control strains even though the input bacteria were similar for all the strains indicating that the knockdown strain had decreased infection ability than the control strains (Fig. 3B). Further, to verify this, confocal microscopy study was performed by infecting THP-1 cells with fluorescein isothiocyanate (FITC)-labeled *M. tb* strains (*M. tb* H37Rv, *M. tb* pSD5A37.1mod/dapB-AS, and vector control) and percent infection was determined as described in Materials and Methods. It was observed that the knockdown mutant strain exhibited a reduced rate of infection as early as after 1 hour of infection as compared with wild-type and vector control strains. The infection rates achieved for the wild type and vector control were 89% and 62%, respectively, whereas the knockdown mutant strain exhibited 32% infection (Fig. S3). Moreover, the infection rate was followed for 4 hours and the infection rate of the knockdown strain was still much reduced than that of the control strains. Taken together, these observations suggested that DapB knockdown mutant certainly has reduced ability to infect macrophages.

Furthermore, on subsequent days of infection, *M. tb* pSD5A37.1mod/dapB-AS mutant displayed a significantly impaired intracellular growth inside the macrophages as compared with *M. tb* H37Rv and *M. tb* vector control, which showed a progressive increase in the growth at different time points post infection (Fig. 3B). Hence, these results suggested that *dapB* is important for *in vitro* as well as intracellular survival of *M. tb.* The data indicate DapB to be an attractive and important drug target for the identification of inhibitors against *M. tb*.

### Identification of DapB inhibitors by structure-based virtual screening against the active site of DapB

**Fig 4:**
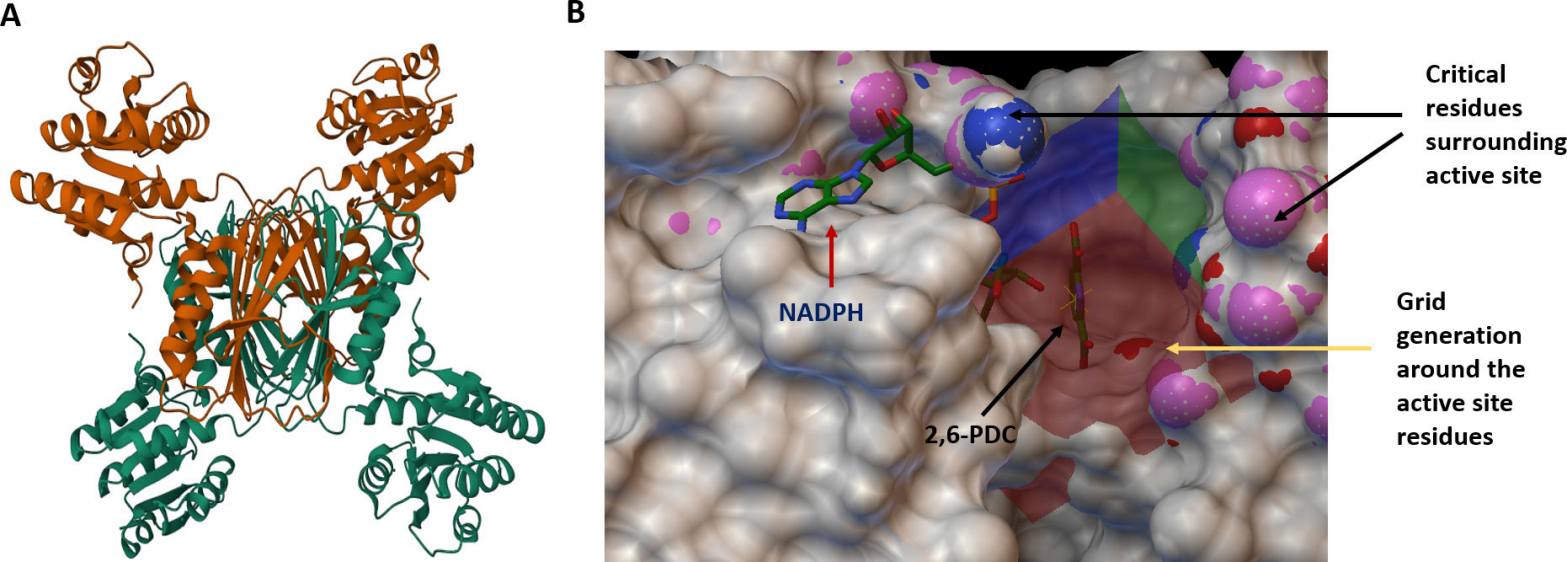
Crystal structure of DapB and docking sites employed for virtual screening. (**A**) The figure shows the three-dimensional structure of DapB (PDB ID: 1YL5) in its apo form. (**B**) Virtual screening was carried out against the active site of DapB. The critical residues around the active site such as Glu-130, His-132, His-133, Lys-136, Asp-138, Ala-139, Pro-140, Ser-141, Gly-142, Thr-143, and Ala-144 are highlighted as colored surface and were used for grid preparation for performing docking. NADPH (cofactor of DapB) and 2, 6-PDC (substrate analog) are shown by red and black colored arrows, respectively. Figure is prepared by using Autodock4.2.

Our data validated that DapB is required for the growth of *M. tb* both *in vitro* and inside macrophages; thus, we attempted to identify DapB inhibitors by employing *in silico*-based virtual screening. For performing structure-based inhibitor identification studies, we employed a library of 260,071 molecules from NCI Open Database that was filtered for drug likeness on the basis of Lipinski’s rule of five (26). This filtered library of 95,748 compounds was employed for carrying out *in silico* virtual screening by using AutoDock 4.2 (27). The three-dimensional crystal structures of *M. tb* DapB were available in the PDB database in either its apo form or NADH/NADPH bound form. These structures provide detailed insight into the crucial residues involved in cofactor/substrate binding (18, 19). Figure. 4A shows a three-dimensional structure of DapB in its apo form (PDB ID- 1YL5) which was selected for virtual screening (19). Based on the amino acid sequence alignment of DapB from several organisms, a region known as “DAP box” with the sequence 130′ELHHXXKXDAPSGTA′144 was found to be conserved among various bacteria which could be involved in substrate binding (9). Thus, these residues neighboring the active site (Glu-130, His-132, His-133, Lys-136, Asp-138, Ala-139, Pro-140, Ser-141, Gly-142, Thr-143, and Ala-144) were selected for grid generation and docking of the compounds (Fig. 4B). Additionally, the grid was centered at an area where 2,6-pyridinedicarboxylic acid (2,6-PDC), which is a known substrate analog and a non-specific inhibitor, is seen to be bound in the crystal structure of *M. tb* DapB (PDB ID- 1YL5) (18). Further, the structure of 2,6-PDC was extracted from PubChem and docked at the grid site to confirm its binding as highlighted in Fig. 4B. It was seen that 2,6-PDC docked well at the active site and was surrounded by the abovementioned crucial residues, validating our docking parameters. Docking was performed at the selected grid by employing the default parameters of autodock4.2. After docking, molecules were shortlisted based on their docking scores that indicated their free energy of binding (∆G). The top molecule in our study had a docking score of −10.45, and 60 compounds with the docking score in the range of −10.45 to −8.59 were procured from NCI-DTP based on their availability for carrying out further experiments.

### Inhibitory potential of the compounds against DapB activity

**Fig 5:**
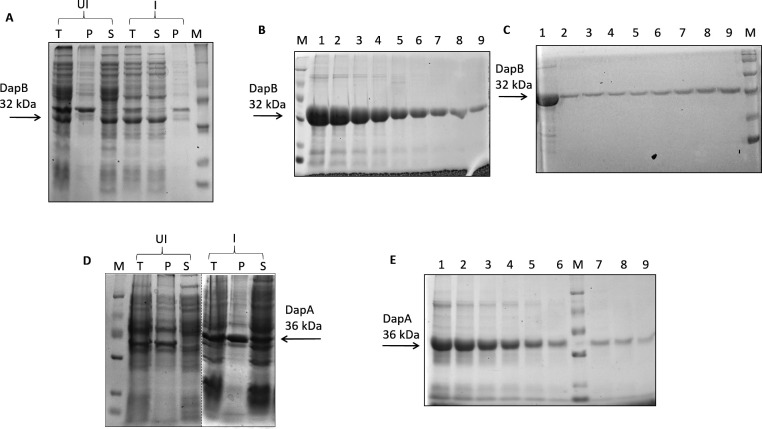
Expression and purification of recombinant proteins (DapB and DapA). (**A**) Dihydrodipicolinate reductase expression was induced with 0.5  mM IPTG (isopropyl-β-d-thiogalactopyranoside) at 37°C for 3 hours. (**B**) 6X-His tagged recombinant DapB was purified by Ni-NTA affinity chromatography. Eluted protein fractions were loaded on 12.5% polyacrylamide gel (lanes 1–9). (**C**) Ni-NTA purified DapB (lane 1) was loaded on a Sephadex G-200 gel filtration column, and eluted fractions (lanes 2–9) were electrophoresed on 12.5% polyacrylamide gel. (**D**) Expression of DapA was induced with 0.5 mM IPTG at 18°C, overnight. Dashed line represents that sections from different gel pictures have been brought together. (**E**) Figure shows eluted fractions (lanes 1–9) of 6X-His tagged recombinant DapA purified by Ni-NTA affinity chromatography. M denotes protein molecular weight marker (from the top: 97, 66, 43, 29, 20, and 14 kDa). For Fig. 5A and D, UI, uninduced sample; I, induced sample; T, total cell lysate; P, pellet; S, supernatant.

For the evaluation of the inhibitory potential of the top-ranking compounds obtained after virtual screening against the enzymatic activity of *M. tb* DapB, we first standardized the DapB enzymatic assay. For this, *M. tb dapB* and *dapA* genes were cloned in pET28a vector. The recombinant positive clones of pET28a/dapB and pET28a/dapA were screened by restriction digestion that resulted in a fall out of approximately 700 bp and 900 bp, respectively, and were further confirmed by sequencing (Fig. S4). We further analyzed the expression and localization of the recombinant proteins (Fig. 5A and D). It was observed that both the proteins were expressed in soluble fraction of the cell and were then subsequently purified by Ni-NTA affinity chromatography (Fig. 5B and E). DapB was further purified by gel filtration chromatography for obtaining pure protein for raising antibodies (Fig. 5C). The purified enzymes were employed to determine the kinetic properties of the starting substrates ASA and sodium pyruvate. The K_m_ values of ASA and sodium pyruvate were found to be 345 µM and 125 µM, respectively (Fig. S5).

**Fig 6:**
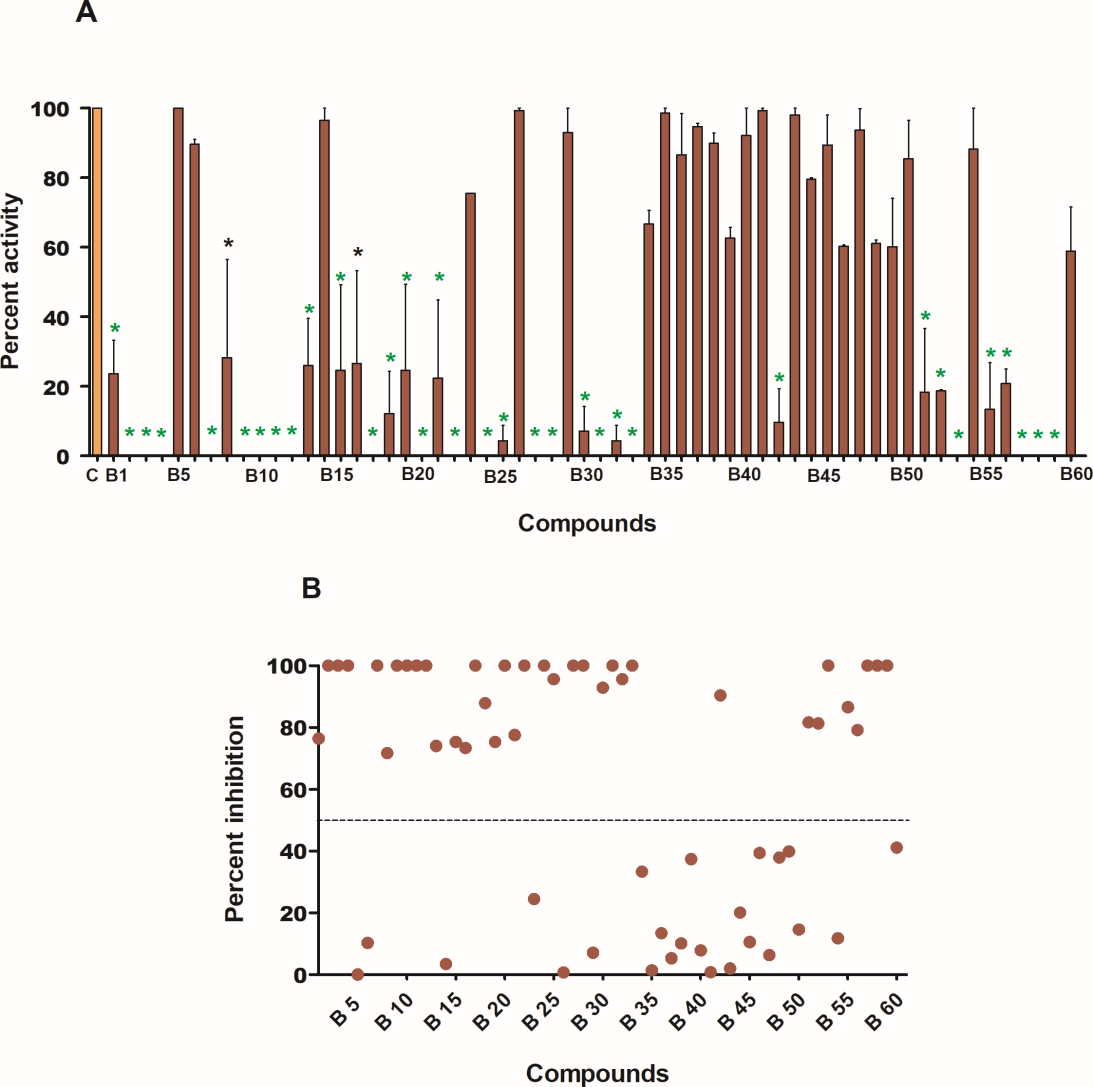
Evaluation of inhibitory potential of the compounds against enzymatic activity of DapB. (**A**) Compounds were screened against DapB activity by using a standardized coupled biochemical assay at a concentration of 100 µg/mL. Bar graph represents percent activity in the presence of inhibitors (brown-colored bars). Percent activity in the control (without inhibitor) is considered as 100% and is represented by orange-colored bar. The data are represented as the mean ± SEM (error bars) of at least two independent experiments. Black-colored stars represent *P* < 0.01, and green-colored stars represent *P* < 0.001 by one-way ANOVA, Tukey’s multiple comparison test. (**B**) Scatter plot of all the compounds showing percent inhibition of DapB enzymatic activity at a concentration of 100 µg/mL (derived from the percent activity data given in 6A). Each dot represents mean percent inhibition of two independent experiments. Thirty-six compounds showed more than 50% inhibition (denoted by dashed line) of DapB activity.

For the evaluation of the inhibitory potential of the top-ranking compounds against the enzymatic activity of DapB, 60 compounds procured from NCI-DTP were initially screened at a fixed concentration of 100 µg/mL wherein 36 compounds exhibited more than 50% inhibition of DapB activity as shown in Fig. 6; Table 1. Moreover, 29 compounds (B2, B3, B4, B7, B9, B10, B11, B12, B17, B18, B20, B22, B24, B25, B27, B28, B30, B31, B32, B33, B42, B51, B52, B53, B55, B56, B57, B58, and B59) exhibited greater than 80% inhibition of the enzyme activity (Table 1). All the compounds with greater than 50% enzyme inhibition at 100 µg/mL were then screened at varying concentrations of the compounds ranging from 10 µg/mL to 100 µg/mL for the determination of their IC_50_ values. Fifteen compounds, namely, B2, B7, B9, B10, B12 B17, B20, B22, B27, B28, B33, B52, B55, B58, and B59 displayed IC_50_ of less than 20 µg/mL (Table 1). Among all the compounds, B27 was found to be the most potent enzyme inhibitor with an IC_50_ value of 4.4 µg/mL followed by B2 and B17 with IC_50_ of 5 µg/mL and 6 µg/mL, respectively (Table 1).

**TABLE 1:** List of the compounds that inhibited enzymatic activity of DapB at a concentration of 100 µg/mL and their respective IC_50_ values

No.	Compounds	IC_50_ (μg/mL) exhibited by compounds in biochemical assay	No.	Compounds	IC_50_ (μg/mL) exhibited by compounds in biochemical assay
1	B1	82	19	B22	10
2	B2	5	20	B24	28
3	B3	31	21	B25	40
4	B4	25	22	B27	4.4
5	B7	6.6	23	B28	15
6	B8	100	24	B30	35
7	B9	10	25	B31	21.5
8	B10	11	26	B32	61
9	B11	49	27	B33	10
10	B12	10	28	B42	20.9
11	B13	58	29	B51	88.7
12	B15	100	30	B52	15.7
13	B16	100	31	B53	25
14	B17	6	32	B55	18
15	B18	70	33	B56	34
16	B19	100	34	B57	35
17	B20	17.5	35	B58	14
18	B21	98	36	B59	11

### Evaluation of inhibitory potential of the compounds against the *in vitro* growth of *M. tb*

**Fig 7:**
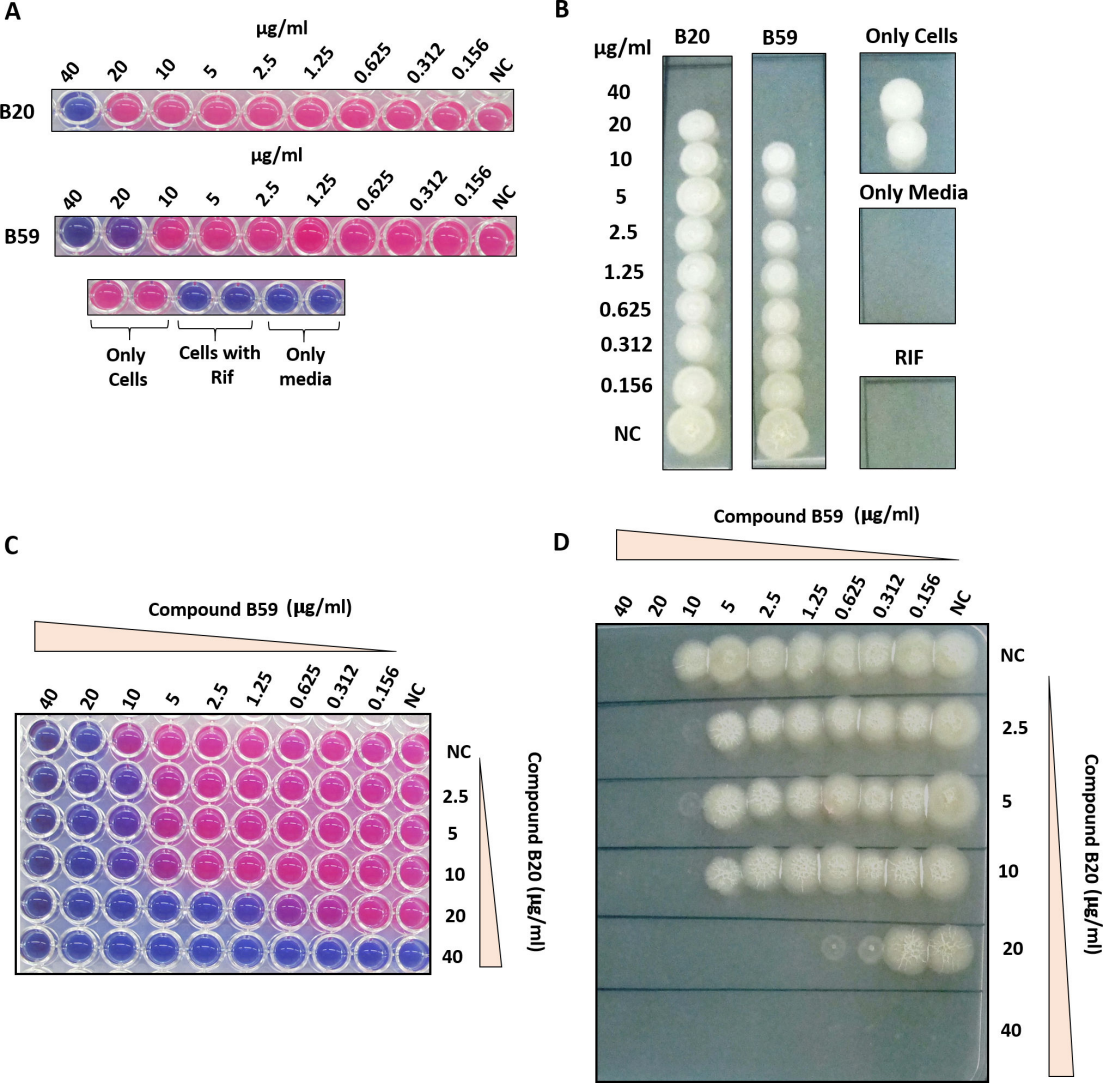
Evaluation of inhibitory potential of the compounds against *in vitro* growth of *M. tb* by resazurin microtiter assay. (**A**) *Mycobacterium tuberculosis* cells were incubated with varying concentrations of compounds (0.156 µg/mL–40 µg/mL) for 7 days at 37°C in a 96-well plate, followed by the addition of resazurin dye and visualization of change in the color of dye. Blue-colored wells indicate non-viable *M. tb* cells whereas pink-colored wells indicate viable mycobacterial cells. Wells containing rifampicin (0.25 µg/mL) and only MB7H9 medium were used as positive and negative controls, respectively. (**B**) MIC_99_ value was determined by spotting an aliquot from each well onto a 7H11 agar plate to check the growth of bacteria. Wells with blue-color dye in Fig. 7A showed no growth on agar plates. NC, well without compound. (**C**) Evaluation of interaction between compounds B59 and B20 by employing checkerboard assay. *Mycobacterium tuberculosis* cells were incubated with varying concentrations of compounds B59 and B20 diluted along the *x*-axis and *y*-axis in a 96-well format for 7 days at 37°C, and the growth of the bacteria was analyzed by resazurin dye-based method. (**D**) MIC_99_ value of each compound in combination with the other compound was determined by spotting an aliquot from each well on to 7H11 agar plates. NC, well without compound. MIC_99_ is considered as the concentration of the well that showed no visible growth on the agar plate.

Further, we evaluated the inhibitory potential of the compounds that exhibited more than 50% inhibition of DapB enzymatic activity at 100 µg/mL against the *in vitro* growth of *M. tb* by employing resazurin dye-based reduction assay (28, 29). Varying concentrations of the compounds in the range of 0.156 µg/mL to 40 µg/mL were incubated with *M. tb* H37Rv cells for 7 days followed by the addition of resazurin dye, and the conversion of the blue color of the dye to a pink color was visualized. Moreover, non-conversion of resazurin to resorufin due to the presence of non-viable cells was confirmed by drop plating an aliquot of the cells from each well onto 7H11 agar plates. Rifampicin was employed as a positive control. Among all compounds, B20 and B59 with IC_50_ values of 17.5 µg/mL and 11 µg/mL, respectively, inhibited the *in vitro* growth of *M. tb* with MIC_99_ values of 40 µg/mL and 20 µg/mL, respectively (Fig. 7A and B; Table 2). Further, CFU enumeration at selected concentrations of the compounds, B20 and B59, clearly showed that even at 0.5X MIC_99_ and 0.25X MIC_99_ values, there is indeed inhibition of the bacterial growth, which was not discernible by resazurin assay and drop plating (Fig. S6). The remaining compounds which showed greater than 50% inhibition of DapB enzymatic activity at 100 µg/mL and did not display any inhibition against *in vitro* growth of *M. tb* in broth culture till 40 µg/mL were further evaluated at higher concentrations; however, none of them exhibited anti-mycobacterial activity even till 100 µg/mL. To summarize, two compounds were identified that possess inhibitory potential against the growth of *M. tb* as well as inhibited DapB enzymatic activity, albeit at varying levels. Further, these two compounds (B59 and B20) were subjected to checkerboard analysis to study their interaction and their ability to potentiate each other’s inhibitory action (Fig. 7C and D). The growth of bacteria in the presence of serially diluted concentrations of compounds B59 and B20 was monitored by resazurin dye-based reduction assay as mentioned in the Materials and Methods section. The results showed that both the compounds B59 and B20 indeed synergize with each other with a ΣFIC value of 0.31. The individual MIC_99_ values of both the compounds changed because of the addition of the other compound resulting in an increase in their inhibitory activities (Fig. 7C and D). It was observed that compound B59, which was earlier showing an MIC_99_ value of 20 µg/mL, now exhibited an MIC_99_ value of 1.25 µg/mL in the presence of 20 µg/mL of compound B20 (Fig. 7C and D). Similarly, the MIC_99_ value of compound B20 increased from 40 µg/mL to 10 µg/mL in the presence of 10 µg/mL of compound B59. Further, fractional inhibitory concentration (FIC) values of the compounds were calculated and found to be 0.062 and 0.25 for compounds B59 and B20, respectively. The ΣFIC value was estimated to be 0.31, which represents synergistic action between these compounds.

**TABLE 2:** List of the compounds that showed inhibition of *M. tb* growth

No.	Compounds	IC_50_ (μg/mL) exhibited by compounds in biochemical assay	MIC_99_ (μg/mL) exhibited by compounds against *M. tb* growth
1	B20	17.5	40
2	B59	11	20

### Cytotoxicity analysis of the compounds against mammalian cell lines

The compounds, B20 and B59, were assessed for their cytotoxicity against various mammalian cell lines such as THP-1 (human monocytic macrophage cell line), HepG2 (hepatocellular carcinoma cell line), and MCF-7 (human adenocarcinoma cell line) by employing resazurin dye-based assay in a dose response manner (1.0 µg/mL–200 µg/mL). Compound B59 exhibited no cytotoxicity till 200 µg/mL in all three cell lines employed in the study. However, compound B20 exhibited a CC_50_ value of 150 µg/mL against THP-1 and HepG2 cell lines, whereas no toxicity was observed against MCF-7 cells till 200 µg/mL. The CC_50_ values were calculated by plotting a graph of fluorescent intensity against various concentrations of compound (Table 3).

**TABLE 3:** CC_50_ values of compounds, B20 and B59, against various mammalian cell lines

No.	Compounds	IC_50_ (μg/mL) exhibited by compounds in biochemical assay	MIC_99_ (μg/mL) exhibited by compounds against *M. tb* growth	(CC_50_ , μg/mL) THP-1	(CC_50_ , μg/mL) HepG2	(CC_50_ , μg/mL) MCF-7
1	B20	17.5	40	150	150	>200
2	B59	11	20	>200	>200	200

### Evaluation of the ability of compounds to inhibit intracellular growth of *M. tb* inside macrophages

**Fig 8:**
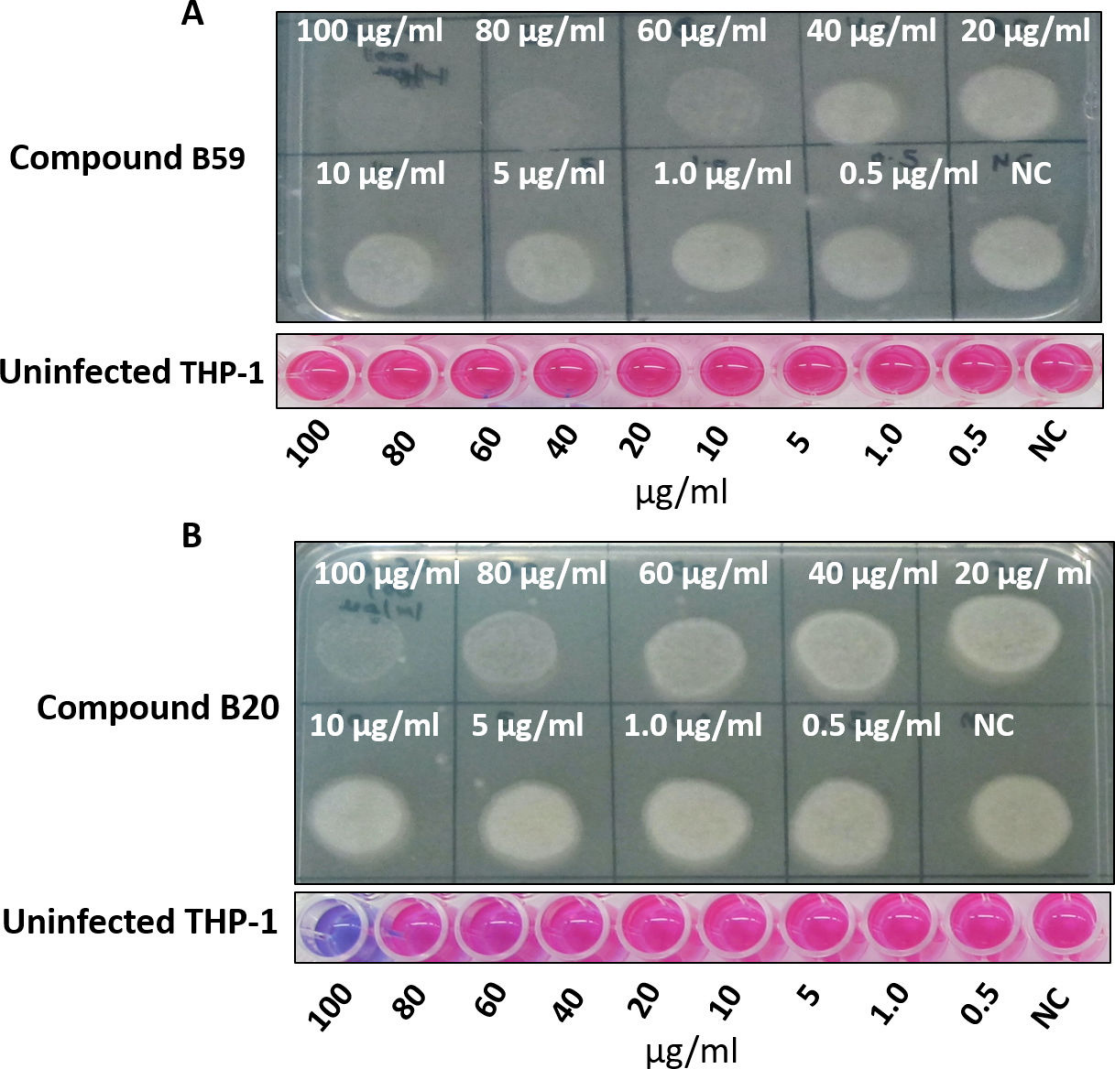
Evaluation of the ability of compounds (B59 and B20) to inhibit the intracellular growth of the pathogen. Phorbol 12-myristate 13-acetate (PMA)-activated THP-1 macrophages were infected with *M. tb* H37Rv, and the growth of pathogen in the presence of varying concentrations of compounds was visualized on agar plates after 5 days of infection. (**A**) Compound B59 displayed inhibition of *M. tb* growth at 60 µg/mL and above. The bottom panel depicts cytotoxicity exhibited by compound B59 toward macrophages by resazurin microtiter dye. Compound B59 did not display any cytotoxicity toward the macrophages. (**B**) Compound B20 displayed inhibition of *M. tb* growth at a concentration of 80 µg/mL and above. It exhibited toxicity toward THP-1 macrophages at a concentration of 100 µg/mL. NC, well without compound.

Mycobacteria are an intracellular pathogen and reside within host macrophages; thus, it is important to evaluate the inhibitory potential of the compounds to inhibit the growth of intracellular *M. tb* residing inside macrophages. Based on the results obtained, two compounds (B20 and B59) that inhibited DapB activity as well as growth of bacteria *in vitro* were further evaluated for their ability to inhibit the growth of intracellular *M. tb* inside macrophages as described in experimental procedures. For this, activated THP-1 macrophages were infected with *M. tb* H37Rv at an MOI of 1:5 and the growth of intracellular bacteria was assessed in the presence of varying concentrations of compounds by carrying out CFU analysis. Rifampicin was taken as a positive control. It was observed that compound B59 inhibited the growth of intracellular pathogen till a concentration of 60 µg/mL as no growth was observed on agar plate at this concentration; however, less growth was visualized at a concentration of 40 µg/mL as shown in Fig. 8A. Moreover, B59 did not display any toxicity toward THP-1 cells at any of these concentrations employed in the study (Fig. 8A, lower panel). Additionally, percentage inhibition of *M. tb* growth at each concentration was determined by CFU analysis by plating serially diluted samples on agar plates. It was observed that compound B59 exhibited an intracellular MIC_90_ value of 73 µg/mL (Fig. S7A). In the case of compound B20, there were very few pinpointed colonies at a concentration of 100 µg/mL and less growth was seen at 80 µg/mL concentration; however, conspicuous growth was observed at lower concentrations as shown in Fig. 8B. Moreover, compound B20 showed toxicity toward THP-1 cells at a concentration of 100 µg/mL as indicated by a blue-colored well after addition of resazurin dye (Fig. 8B, lower panel), although it did not display any toxicity at lower concentrations (Fig. 8B, lower panel). Furthermore, CFU enumeration for compound B20 displayed an MIC_90_ value of 95 µg/mL (Fig. S7B). Hence, our results suggested compound B59 as a hit molecule which exhibited inhibitory potential against the DapB activity and inhibited the *in vitro* as well as intracellular growth of *M. tb*. Compound B59 also had no significant cytotoxicity against various mammalian cell lines.

**Fig 9:**
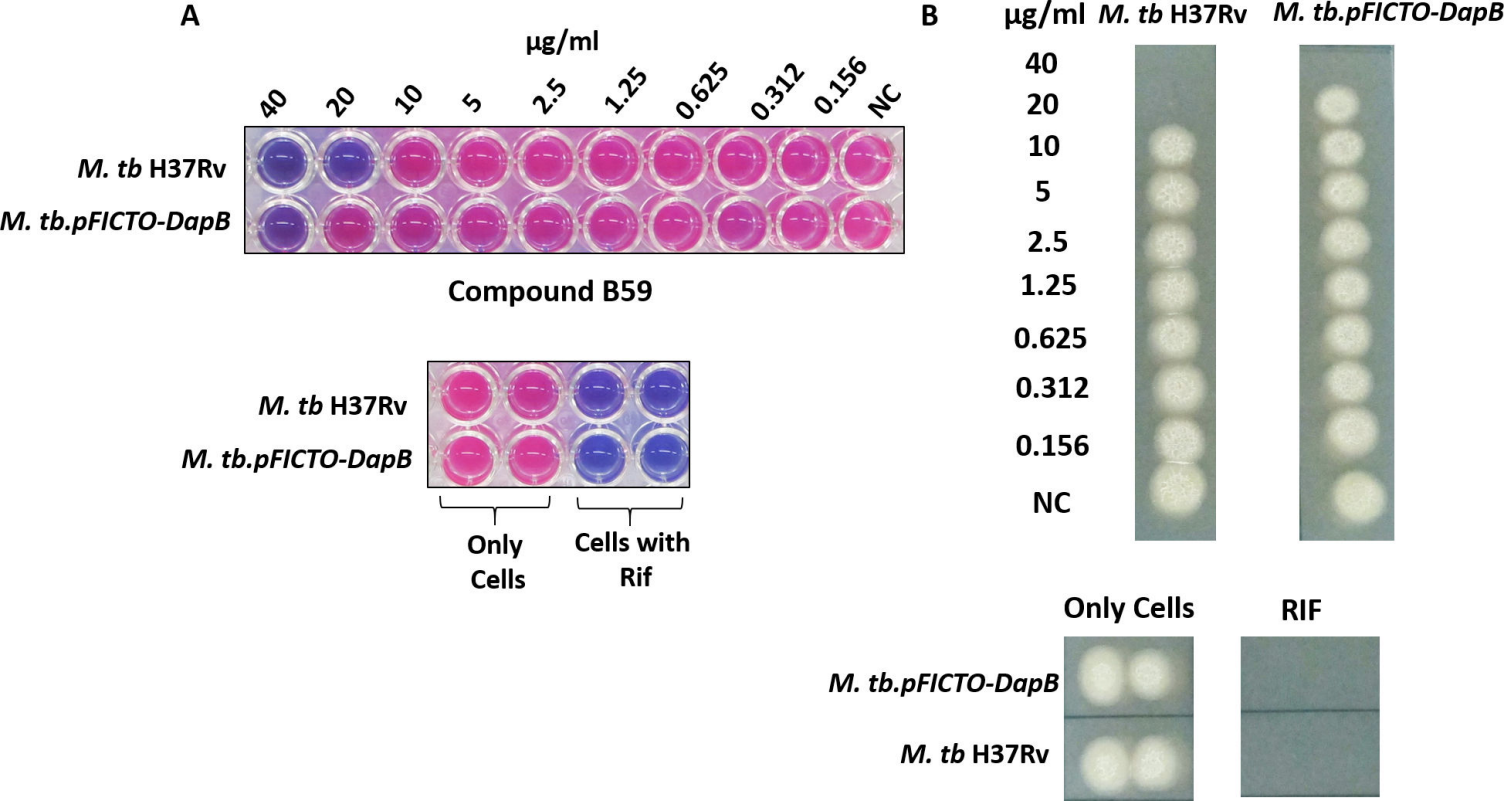
Evaluation of the target specificity of compound B59 by employing resazurin dye reduction assay. (**A**) Varying concentrations of compound B59 (0.156 µg/mL to 40 µg/mL), along with appropriate controls, were incubated with various mycobacterial strains (*M. tb H37Rv* and *M. tb.pFICTO-dapB*) for 7 days at 37°C followed by the addition of resazurin dye and visualization of change in the color of dye. Blue-colored wells indicate non-viable *M. tb* cells whereas pink-colored wells indicate viable mycobacterial cells. (**B**) MIC_99_ value was evaluated by spotting an aliquot from each well onto agar plates to assess the growth of bacteria.

Additionally, to confirm DapB as the intracellular target of B59, a DapB-overexpressing strain, *M. tb.pFICTO-dapB*, was employed that produces additional DapB protein fused to 3x-flag tag. To verify the overexpression of DapB in the overexpression strain, lysates of the wild-type *M. tb* and *M. tb.pFICTO-dapB* were assessed by immunoblotting using anti-DapB antibody. We observed two bands (~32 kDa and ~35 kDa) in the case of *M. tb.pFICTO-dapB*, one corresponding to DapB and the other to flag-tagged DapB while the wild-type *M. tb* had only the DapB band (Fig. S8). It was observed that the cumulative level of DapB was ~35% higher in *M. tb.pFICTO-dapB* as compared with the wild-type strain after normalization with the loading control. For the determination of the MIC_99_ value, varying concentrations of compound B59 (0.156 µg/mL to 40 µg/mL) were incubated with *M. tb H37Rv* and *M. tb.pFICTO-dapB* strains and inhibition was evaluated as described above for the *M. tb in vitro* assays. It was observed that the MIC_99_ value of compound B59 increased to 40 µg/mL (2x MIC_99_) in the overexpressing strain as compared with 20 µg/mL in the wild-type strain, suggesting DapB as a target for compound B59 inside *M. tb* cells (Fig. 9A and B).

### Identification of the binding interactions of the hit compound with DapB

**Fig 10:**
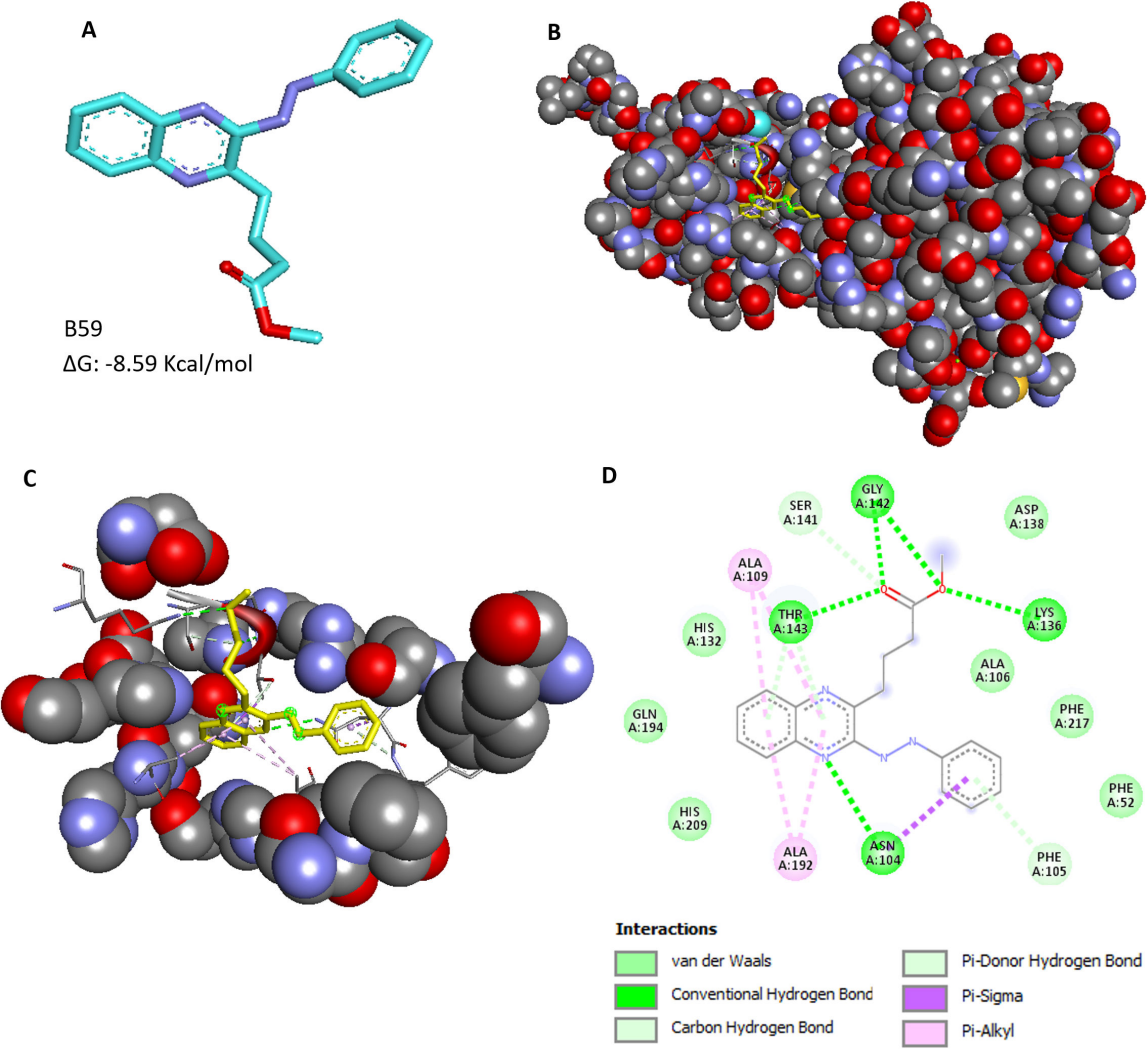
Binding mode analysis of the hit compound, B59. (**A**) Chemical structure of the compound B59, 4-(3-Phenylazoquinoxalin-2-yl) butanoic acid methyl ester (cyan: carbon, purple: nitrogen, and red: oxygen). (**B**) Figure displays binding of compound B59 at the active site of DapB. (**C**) Pocket residues of receptor interacting with the ligand, B59. (**D**) Key interactions of compound B59 with the residues of receptor. The receptor-ligand interaction diagrams were generated by using BIOVIA Discovery Studio 2021.

Based on the DapB enzyme inhibition assays, *in vitro* MIC studies, intracellular inhibition assays, and cytotoxicity experiments, compound B59 (NSC-703161), namely, 4-(3-Phenylazoquinoxalin-2-yl) butanoic acid methyl ester, was identified as a hit molecule; hence, the probable binding mode of this compound at the active site of DapB was predicted (Fig. 10A). It was observed that compound B59 docked very well at the active site of the enzyme (Fig. 10B and C). The residues Phe 52, Asn 104, Phe 105, Ala 106, Ala 109, His 132, Lys 136, Asp 138, Ser 141, Gly 142, Thr 143, Ala 192, Gln 194, His 209, and Phe 217 were predicted to be interacting with compound B59 (Fig. 10D). It was found that compound B59 interacts with residues Asn 104, Lys 136, Gly 142, and Thr143 by forming hydrogen bonds (Fig. 10D). Compound B59 interacts with residues Ala 109 and Ala 192 via the π -alkyl bond (Fig. 10D). It also exhibited Van der Waals interactions with residues Phe 52, Ala 106, His 132, Asp138, Gln 194, His 209, and Phe 217 (Fig. 10D). Moreover, it displayed a carbon hydrogen bond with Ser 141 and a π-donor hydrogen bond with Phe 105 (Fig. 10D). It was observed that residue Asn 104 interacts with compound 59 by forming both hydrogen bonds and hydrophobic interactions like π–σ bond (Fig. 10D). We noted that out of these 15 interacting residues, six residues (His 132, Lys 136, Asp 138, Ser 141, Gly 142, and Thr 143) are known to be involved in the direct binding of the substrate DHDP, and thus, B59 might dock itself by disrupting substrate binding (9). We also found various non-conserved residues (Phe 52, Ala 106, Ala 109, Ala 192, Gln 194, His 209, and Phe 217) to be interacting with B59 which might be involved in providing specificity of the inhibition, which can be investigated in further studies. Hence, it was concluded that compound B59 binds at the active site of DapB by making contacts with several key residues of the protein.

## DISCUSSION

Tuberculosis, caused by *M. tuberculosis,* is one of the most dreadful infectious diseases causing millions of deaths every year. Despite the availability of effective anti-TB drug regimen, non-adherence to the therapy often results in the emergence of multidrug-resistant strains. Thus, there is an urgent need to strengthen the TB drug discovery pipeline by identifying more potent inhibitory molecules that can shorten the chemotherapy period. As a prerequisite for the identification of new inhibitors, it is important to understand the role of crucial and important targets of *M. tb*. Hence, in this study, we have attempted to target an enzyme of *M. tb* DapB, involved in lysine amino acid as well as cell wall biosynthesis. For this, an antisense-based strategy was employed for generating a knockdown mutant strain of *dapB* by cloning the gene in antisense orientation in pSD5 vector under a strong synthetic promoter, A37.1mod (23, 24). We had initially made multiple attempts to knockout the *dapB* gene by homologous recombination; however, we did not succeed in obtaining the mutant colonies suggesting a crucial role of DapB in the *M. tb* growth. Hence, we employed antisense knockdown strategy for the generation of *dapB* knockdown mutant in view of the fact that for essential genes, knocking down their levels will prevent the complete abolishment of the targeted protein synthesis, thus resulting in viable colonies that may, however, be growth defective (30 – 32). We observed colonies for *dapB* knockdown mutant *M. tb* cells that were faint and small in size as compared with the characteristic rough and large size colonies observed in case of the parental strain. This clearly correlates with the suggested role of *dapB* in meso-dap biosynthesis that is an important component of the mycobacterial cell wall, implying that knockdown of *dapB* might have an effect on the morphology and integrity of the cell wall. Moreover, small size of the colonies also suggests the role of DapB in the in *vitro* growth of *M. tb*. We performed immunoblot studies and observed more than 90% reduction in the DapB levels in the antisense knockdown mutant strain as compared with the parental strain, thus, confirming successful construction of *dapB* knockdown mutant strain. Moreover, similar levels of DapB were observed in the wild-type and the vector control strains. Use of antisense strategy indeed leads to significant inhibition in the levels of the targeted protein by affecting the protein translation process, as shown by several investigators and evident from our results also (22, 33, 34). Moreover, we observed a substantial reduction in the DapB levels, which is possibly due to the use of a strong constitutive mycobacterial promoter A37.1mod (23, 24).

Further, to evaluate the role of *dapB* in the survival of *M. tb*, growth kinetics study of the antisense knockdown mutant strain, wild-type *M. tb* strain, and *M. tb* vector control strain was carried out in *in vitro* broth culture. We found that the antisense knockdown mutant strain showed significant growth defect in liquid medium as compared with the parental as well as the vector control strains. This indicates that DapB is crucial for the *in vitro* growth of *M. tb.* The most possible explanation for attenuated growth in DapB knockdown strain is its inability to synthesize meso-DAP and lysine, important constituents of peptidoglycan and amino acid biosynthesis, respectively. A deficiency of these constituents augurs well for an attenuated phenotype. We also assessed the ability of all mycobacterial strains to survive inside THP-1 macrophages at various time points. Although infection of THP-1 macrophages with all the strains was performed with similar inoculum (input bacteria), knockdown mutant strain exhibited significantly reduced CFU after 4 hours (day 0) of infection. This observation suggests that the knockdown strain possibly has reduced ability to infect the macrophages. This was verified by confocal microscopy studies wherein knockdown mutant strain displayed a reduced rate of infection as compared with the parental strain (Fig. S3). This reduced infectivity of knockdown mutant strain might be associated with the role of DapB in cell wall biosynthesis. The inability of the mutant to synthesize meso-DAP can result in defective cell wall integrity that could either affect the bacterial attachment to macrophage surface or reduce its internalization. It would be interesting to further investigate the mechanism of reduced infectivity of this strain separately in future studies. Moreover, at further time points, knockdown mutant grew, albeit at a retarded rate, as compared with the wild-type and vector control strains. Hence, for the first time, we showed the importance of *dapB* in the *in vitro* as well as intracellular growth of *M. tb*.

Our studies showed that DapB is important for the growth of *M. tb*, suggesting it to be a promising target for the discovery of new small molecule inhibitors against mycobacteria. Thus, we performed *in silico* virtual screening against the active site of DapB by using Audock4.2 software. We explored the available crystal structure of DapB (PBD ID: 1YL5) and found a few conserved critical residues surrounding the active site of DapB that were used for grid generation (18). Docking was performed with a filtered library of ~95,000 molecules from the NCI open database, and a highest docking score of −10.45 was observed in our study. Further, we procured 60 molecules for assessing their inhibitory potential against the DapB enzyme. For this, we standardized the enzymatic assay for DapB, which is a coupled assay and required an upstream enzyme DapA. Hence, the genes for both the proteins were cloned in pET28a vector and expressed and the proteins were purified by using Ni-NTA affinity chromatography.

After standardizing the assay, we evaluated the ability of 60 compounds (procured from NCI) to inhibit the enzymatic activity of DapB. We found that 36 compounds exhibited more than 50% inhibition of enzymatic activity of DapB at a concentration of 100 µg/mL, out of which 29 compounds exhibited more than 80% inhibition of DapB activity. Further, we determined the IC_50_ values of these 36 compounds and 15 compounds, namely, B2, B7, B9, B10, B12, B17, B20, B22, B27, B28, B33, B52, B55, B58, and B59 were found to exhibit IC_50_ values of less than 20 µg/mL. The most potent compound, B27, in our study displayed an IC_50_ value of 4.4 µg/mL followed by B2 and B17 with IC_50_ values of 5 µg/mL and 6 µg/mL, respectively. These enzyme inhibitors were further evaluated for their ability to inhibit the *in vitro* growth of *M. tb,* wherein compounds B20 and B59 exhibited mycobacterial growth inhibition. It was noted that the most potent enzyme inhibitors such as B27, B2, and B17 and the remaining enzyme inhibitors failed to inhibit the growth of *M. tb* even at higher concentration. One of the probable reasons for such observation could be their limitation to permeate through the mycobacterial cell wall. Compound B59, that displayed an IC_50_ value of 11 µg/mL, inhibited the growth of *M. tb* with an MIC_99_ value of 20 µg/mL, while compound, B20, that displayed an IC_50_ value of 17.5 µg/mL showed an MIC_99_ value of 40 µg/mL against the *in vitro* growth of *M. tb*. Moreover, these compounds also displayed synergism against the *in vitro* growth of *M. tb* with ΣFIC value of 0.31 highlighting that compounds B59 and B20 potentiate the inhibitory potential of each other when used in combination. In the field of pharmacology, synergistic interaction among drugs is often encountered and is considered highly beneficial to maximize the therapeutic effects of individual drugs and use of lower doses and thus minimize side effects (35). Synergism exhibited by inhibitors can arise due to several factors such as inhibition of the same target protein, increased permeability, membrane disruption, and peptidoglycan damage; all of which might result in an enhanced inhibition (36). A synergistic interaction between compounds B59 and B20 is indeed interesting, which can be explored further along with the determination of a precise mechanism of their synergistic action.

Subsequently, we analyzed the cytotoxicity of these two compounds against various mammalian cell lines such as THP-1, HepG2 (hepatocellular carcinoma cell line), and MCF-7 (human adenocarcinoma cell line) by employing resazurin dye-based assay. Compound B59 did not exert any toxicity against these cell lines till a concentration of 200 µg/mL (maximum concentration employed in the study). However, compound B20 exerted slight toxicity against THP-1 and HepG2 cell lines at a concentration of 150 µg/mL and above, while no toxic effect was seen against MCF-7 cells till 200 µg/mL. Subsequently, we evaluated the ability of these two compounds (B59 and B20) to inhibit the growth of mycobacteria inside macrophages. Compound B59 was shown to inhibit the growth of the pathogen inside THP-1 macrophages with MIC_90_ of 73 µg/mL without exerting any toxic effect against THP-1 cells. Compound B20 inhibited the intracellular growth of *M. tb* with MIC_90_ of 95 µg/mL; however, it displayed cytotoxicity toward THP-1 macrophages at a concentration of 100 µg/mL and above. It is to be noted that we observed differences in the cytotoxicity of compound B20 in two different assays, that is, cytotoxicity assay and intracellular inhibition assay, which is apparently due to the differences in incubation time of these experiments. We believe that a longer incubation of the THP-1 cells with the compound in the intracellular inhibition assay might be responsible for compound B20 to show cytotoxicity at a lower concentration of 100 µg/mL, which is logical in view of the fact that non-specific cytotoxicity might increase with prolonged incubations. We also observed that there was a difference in the efficacy of the compounds against the *in vitro* and the intracellular growth of the pathogen, which could be possibly due to the several factors such as reduced effective intracellular concentration of the compounds inside macrophages or poor permeability inside macrophages since mycobacterial permeability and host cell permeability may vary for different compounds that may depend upon their chemical nature.

Hence, we identified a hit molecule, B59, namely 4-(3-Phenylazoquinoxalin-2-yl) butanoic acid methyl ester belonging to quinoxaline scaffold that inhibited the enzymatic activity of DapB, showed inhibitory potential against the growth of *M. tb* with negligible cytotoxicity, and also showed interactions with key important substrate-binding residues of DapB. It was interesting to note that quinoxaline derivatives have been earlier shown as promising candidates for the development of anti-TB molecules (37 – 42). Further, we attempted to investigate the intracellular target specificity of compound B59 by employing a DapB-overexpressing strain. We showed that there was a two-fold increase in the MIC_99_ value of compound B59 against DapB-overexpressing strain as compared with the wild-type *M. tb* strain. This indeed suggests that DapB is an intracellular target of compound B59 as overexpression of the target DapB resulted in reduced susceptibility of this strain to inhibition by this compound.

In conclusion, our study explored the role of DapB in the growth of *M. tb* and showed that knockdown of *dapB* negatively impacts the ability of *M. tb* to grow *in vitro* as well as inside macrophages. Further, we employed *in silico* virtual screening for the identification of small molecule inhibitors against DapB. The most potent inhibitory molecule in our study is B59, which is a quinoxaline derivative that has not been reported previously. Further studies are, however, required for the lead optimization of B59 to identify and design more potent inhibitory molecules against DapB. To the best of our knowledge, this is the first report showing the importance of *M. tb dapB* in the survival of the pathogen, thus, making it a promising target for drug discovery against *M. tb*.

## MATERIALS AND METHODS

### Bacterial strains

The *M. tb* strains (*M. tb* H37Rv and GFP (green fluorescent protein) expressing *M. tb* H37Rv) (24) were grown in Middlebrook (MB) 7H9 broth medium supplemented with ADC (albumin-dextrose-catalase), 0.5% glycerol, and 0.2% tween 80 at 37°C with constant shaking of 200 rpm or on MB 7H11 agar supplemented with OADC (oleic acid-albumin- dextrose-catalase) and 0.5% glycerol. The mycobacterial shuttle vector, pSD5A7.1mod, containing a kanamycin resistance cassette and a strong synthetic promotor (A37.1mod) was used for *dapB* antisense cloning in *M. tb* (23). The strains of *E. coli* including XL1-Blue and BL21(λDE3) cells were grown in Luria Bertani (LB) broth medium or on LB agar containing kanamycin antibiotic at a concentration of 25 µg/mL.

### Construction and characterization of the antisense construct of dapB (rv2773c) in the *M. tb* H37Rv

The gene encoding *dapB* (dihydrodipicolinate reductase) was amplified by PCR using *M. tb* H37Rv genomic DNA as the template. The forward primer with the sequence -5′CTAGTAACGCGTATGCGGGTAGGCGTCCTTGG 3′ contained the MluI restriction site (dap-F-MluI), and the reverse primer with the sequence -5′ CTAGTACATATGTCAGTGCAGATCGAGTAGGG3′ contained the NdeI restriction site (dap-R-NdeI). The primers were designed in a manner which will result in cloning of the *dapB* gene in antisense orientation downstream to the A37.1mod promoter in pSD5 vector. The amplified DNA product was digested with MluI and NdeI to create cohesive ends and was ligated into vector pSD5A37.1mod digested with the same restriction enzymes. The *E. coli* XL1-Blue cells were transformed with the ligation mixture followed by selection of recombinant cells on kanamycin. The positive clones were screened by restriction digestion by using NdeI and MluI restriction enzymes and confirmed by sequencing. The antisense construct pSD5A37.1mod/dapB-AS was then employed for electroporation of *M. tb* H37Rv cells. Recombinant *M. tb* cells containing the plasmid pSD5A37.1mod/dapB-AS were selected on the basis of kanamycin resistance. As a vector control, pSD5A37.1mod (empty vector) was also employed for electroporation of *M. tb* H37Rv cells. The colonies obtained on kanamycin containing MB7H11 agar plates were patched and further grown in supplemented MB7H9 medium in the presence of kanamycin. Screening of the recombinants containing pSD5A37.1mod/dapB-AS or empty vector was carried out by isolating plasmid DNA from the culture followed by PCR reaction by employing appropriate primers.

### Immunoblot analysis

The bacterial strains were grown till mid-logarithmic phase, and protein lysate was prepared. Briefly, cells were harvested by centrifugation at 4,500 rpm for 10 minutes and were resuspended in 1× phosphate buffer saline (PBS), pH-7.5 containing 1 mM PMSF. The lysate was prepared by bead beating using 0.1 mm zirconia beads and was subjected to centrifugation at 14,000 rpm for 20 minutes at 4°C. The clear supernatant was filtered through a 0.2-µm syringe filter followed by protein estimation. Following that, 70 µg of protein lysate from different strains was separated by SDS-PAGE (12.5%) and immunoblotting was performed by using DapB-specific antibody. *Mycobacterium tuberculosis* FadD13, a mycobacterial cytosolic protein unrelated to the DAP pathway, was employed as a loading control. Anti-FadD13 primary antibody was employed for detection of FadD13 (~54 kDa). Percentage inhibition of DapB levels in *M. tb* vector control and antisense knockdown mutant strain was calculated by comparing the DapB levels in each of the strain to that of *M. tb* H37Rv after normalizing the data with FadD13 levels (loading control). Quantification of the protein levels in the immunoblot was performed by using ImageJ software.

### Growth kinetics study

*Mycobacterium tuberculosis* H37Rv, *M. tb* pSD5 A37.1mod/dapB-AS, and *M. tb* pSD5A37.1mod strains were grown in supplemented MB7H9 medium at 37°C with constant shaking at 200 rpm. The growth of the strains was monitored at regular time points by CFU analysis by plating serial dilutions of the samples on 7H11 agar plates. The growth kinetics was analyzed by plotting the growth curve using GraphPad Prism. The data are represented as the mean ± SEM (error bars) of at least two independent experiments. (*P < 0.05, (**P < 0.01, and ***P < 0.001, two-way ANOVA, Bonferroni post-tests).

### Infection of THP-1 macrophages and bacterial enumeration

The exponentially growing cells of bacterial strains (*M. tb* H37Rv*, M. tb* pSD5A37.1mod/dapB-AS, and *M. tb* pSD5A37.1mod) were syringe passaged to make a single cell suspension and were diluted to the desired OD (OD_600_ of 0.5 corresponds to 3 × 10^7^ CFU/mL). THP-1 (human monocytic cell line) cells were grown in RPMI media reconstituted with 10% FBS and antibiotic-antimycotic mix (ABAM). 1 × 10^5^ cells were seeded in a 96-well flat bottom plate in RPMI media containing 10% FBS and 20 ng/mL phorbol 12-myristate 13-acetate for 16 hours followed by the removal of the media. The PMA-activated macrophages were then infected with different bacterial strains at an MOI of 1:5 (macrophage: bacteria) for 4 hours at 37°C in the presence of 5% CO_2_. Subsequently, amikacin (200 µg/mL) treatment was carried out to kill extracellular bacilli. At various time points (days 0, 2, 4, 6, and 8), cells were lysed with 0.025% SDS solution for 10 minutes and appropriate dilutions of samples were plated on MB7H11 agar plates for CFU enumeration. To rule out any possibility of this SDS treatment on the viability of the DapB knockdown strain, which might have an altered cell wall integrity, it was separately evaluated that this SDS treatment had no specific killing effect on the knockdown strain (Fig. S9).

### Confocal microscopy studies for measuring the rate of intracellular infection

Different *M. tb* strains were grown to A_600nm_ of 0.8 and labeled with FITC. For this, cultures were harvested, washed twice with 0.5 M sodium carbonate-bicarbonate buffer (pH 9.5), and resuspended in the same buffer containing 100 µg/mL FITC followed by an overnight incubation at 4°C. Further, the bacterial cells were harvested at 4500 rpm for 5 minutes, washed twice with PBS, and syringe passaged to make a single cell suspension. For infection, THP-1 cells were seeded at a density of 5 × 10^5^ macrophages per well on poly-L-lysine-coated glass coverslips within a 12-well plate. The infection was performed at an MOI of 1:5 (macrophage: FITC-labeled bacteria) as described above. After infection, the cells were washed once with RPMI media reconstituted with 10% FBS and fixed with 4% paraformaldehyde in PBS. Afterwards, coverslips were mounted by using Prolong Gold antifade reagent and cells were visualized by using a Leica TCS SP5 confocal laser scanning microscope. Subsequently, the percent infection of FITC-labeled bacteria of different *M. tb* strains was determined by analyzing 8–10 fields of infected macrophages. The number of intracellular bacteria was counted for each strain in ~150–200 macrophages. The percent infection was calculated by the formula—(number of intracellular bacteria/number of macrophages) × 100.

### Structure-based virtual screening

The three-dimensional crystal structure of *M. tb* DapB in its apo form (1YL5) was downloaded from RCBS Protein Data Bank (19). Docking was performed by using Autodock4.2, which includes the Lamarckian genetic algorithm at default parameter (27). The protein molecule was prepared by deletion of water molecules, addition of hydrogen atoms, and merging non-polar charges. Virtual screening was carried out by employing a library of 95,748 compounds belonging to NCI open database, which was filtered on the basis of Lipinski’s rules for drug likeness (26). After docking, the compounds were sorted on the basis of their decreasing free binding energies and 60 compounds (denoted as B1-B60) were procured from the National Cancer Institute-Developmental Therapeutics Program (NCI-DTP) based on their availability.

### Cloning and expression of *dapB*

The *M. tb dapB* gene was amplified by PCR using the genomic DNA of *M. tb* H37Rv as the template. Forward primer -5′GTACTAGGATCCCATATGATGCGGGTAGGCGTCCTTGG-3′ containing BamHI and NdeI restriction sites and reverse primer -5′GTACTAAAGCTTCTCGAGTCAGTGCAGATCGAGTAGGG-3′ containing HindIII and XhoI restriction sites were employed. The amplified PCR product was digested with restriction enzymes NdeI and XhoI and subsequently cloned into *E. coli* pET28a expression vector predigested with the same restriction enzymes. The *E. coli* XL1-Blue cells were transformed with the ligation mixture, and the recombinants were selected on kanamycin. Positive clones were screened by restriction digestion of the plasmid DNA isolated from the colonies with NdeI/XhoI, and the construct was confirmed by sequencing. For performing expression and purification studies, *E. coli* BL21 (λDE3) cells were transformed with the construct *pET28a/DapB* and the transformants were grown in LB media till log phase. The expression of DapB was induced by the addition of 0.5 mM isopropyl-1-thio-β-d-galactopyranoside to the culture at 37°C for 3 hours. After induction, cells were harvested by centrifugation at 6,000 rpm for 10 minutes at 4°C. The cellular localization of the recombinant protein was assessed by electrophoresis on 12.5% SDS-PAGE.

### Purification of DapB

The purification of DapB containing N- terminal 6X-His tag was performed by employing Nickel-Nitrilotriacetate (Ni-NTA) affinity chromatography. Briefly, IPTG-induced cells were harvested and resuspended in 25 mL lysis buffer [20 mM Tris-HCl (pH-8.0), 500 mM NaCl, 10 mM imidazole, 1 mM phenyl methyl sulfonyl fluoride (PMSF), 2 mM β-mercaptoethanol, and 5% (vol/vol) glycerol]. The resuspended cells were lysed by sonication followed by centrifugation at 15,000 rpm for 45 minutes at 4°C. The clear lysate was then incubated with 2 mL of Ni-NTA resin pre-equilibrated with equilibration buffer [20 mM Tris-HCl (pH-8.0), 500 mM NaCl, 5 mM imidazole, 2 mM β-mercaptoethanol and 5% (vol/vol) glycerol] for 1 hour at 4°C in an end-on rotator. The flow through was collected, and resin was washed with 30 mL of each wash buffer-1 (equilibration buffer containing 10 mM imidazole), wash buffer-2 (equilibration buffer containing 20 mM imidazole), and wash buffer-3 (equilibration buffer containing 50 mM imidazole). Thereafter, the bound protein was eluted as 1.5 mL fractions by using elution buffer (equilibration buffer containing 250 mM imidazole). The eluted protein fractions were quantified by Bradford estimation assay and analyzed by SDS-PAGE on a 12.5% gel. This affinity-purified protein was employed for activity assay. Further purification of this protein was carried out for the purpose of raising DapB-specific antibodies. For this, Ni-NTA affinity-purified protein was concentrated by using an Amicon Ultra protein concentrator with a 30-kDa filter. The concentrated protein was then further purified by gel filtration chromatography by using a Sephadex G-200 column employing 50 mM Tris, pH 8.0, 150 mM NaCl, and 10% glycerol as the buffer for purification. The eluted fractions were further analyzed by electrophoresis on 12.5% SDS-PAGE.

### Cloning, expression, and purification of DapA

The cloning and expression of *dapA* (dihydrodipicolinate synthase) were performed by using a similar strategy as described above for *dapB* with some modifications. Briefly, the *M. tb dapA* gene was amplified by PCR by using *M. tb* H37Rv genomic DNA as the template. Forward primer -5′ gtactaggatcccatatgtatgatgcgggtaggcgtccttgg-3′ containing NdeI and BamHI restriction sites and the reverse primer -5′gtactaaagcttctcgagtcagtgcagtcgagtaggg-3′ having HindIII and XhoI restriction sites were employed. Further, the digested PCR product was cloned into pET28a vector to create pET28a/dapA recombinant construct as described above. The expression of *dapA* was performed by induction of *E. coli* BL21 (λDE3) cells transformed with pET28a/DapA with 0.5 mM IPTG at 18°C overnight. The DapA enzyme was purified by employing Ni-NTA affinity chromatography as described above, and purified protein was stored at −80°C.

### Dihydrodipicolinate reductase-coupled enzymatic assay

The DapB enzymatic assay was performed as described earlier by Paiva et al. with some modifications (20). The enzymatic assay of DapB is a coupled assay which requires the upstream enzyme DapA. The formation of tetrahydrodipicolinate by DapB was measured by monitoring conversion of NADPH to NADP^+^ spectrophotometrically at 340 nm. The substrate of DapB, i.e., 2,4-dihydrodipicolinic acid, was prepared enzymatically through condensation of Aspartate Semialdehyde [(S)-ASA] and sodium pyruvate by DapA. The standard assay was performed in 50 mM sodium phosphate buffer (pH 7.5) consisting of 400 µM (S)-ASA, 160 µM NADPH, 500 µM sodium pyruvate, 3 µg DapB, and 5 µg DapA in a total reaction volume of 100 µL. The assay components such as sodium pyruvate, NADPH, DapA, and DapB were added in a 96-well flat bottom plate and incubated for 10 minutes at 37°C followed by the addition of (S)-ASA to start the reaction. The decrease in the absorbance of NADPH corresponding to the consumption of substrate was monitored at 340 nm after incubation for 10 minutes at room temperature. The assay mixture without (S)-ASA was utilized as a negative control. The activity of DapB in the presence of compounds was measured similarly; however, the enzyme was pre-incubated with the compounds for 10 minutes at 37°C. The percent activity for the samples in the presence of inhibitors was calculated by using a two-step method. Firstly, the absorbance values at 340 nm for each test sample (with or without the inhibitor) were normalized with the negative control (no substrate control). Subsequently, the percent activity was calculated by using the formula (normalized A_340nm_ of inhibitor sample/normalized A_340nm_ of no inhibitor sample) × 100. Percent inhibition was calculated as 100 minus percent activity.

All the compounds were dissolved in DMSO and were initially screened at a concentration of 100 µg/mL followed by a dose-dependent assay at varying concentrations of compounds (10–100 µg/mL). The IC_50_ value (defined as the concentration of compound which displayed 50% inhibition of enzyme activity) was calculated by plotting a graph of compound concentration versus percent inhibition.

### *Mycobacterium tuberculosis* whole cell growth inhibition assay by Resazurin microtiter assay

The logarithmic phase culture of *M. tb* H37Rv was diluted to a final A_600nm_ of 0.02 (~2 × 10^6^ CFU/mL) and incubated with varying concentrations of the compounds (0.156 µg/mL–40 µg/mL) in a 96-well U-bottom plate for 7 days at 37°C. Subsequently, 30 µL of 0.01% resazurin reagent was added to each well and incubated at 37°C for 24 hours. Resazurin is a blue-colored dye, which undergoes a change in its color in response to the metabolic state of a cell. Active cells will reduce the blue-colored resazurin into a pink-colored dye, resorufin, which is highly fluorescent, and its intensity of the fluorescence is directly proportional to the number of living cells. Following that, plates were visualized for any change in the color and the fluorescence of each well was measured at an excitation/emission wavelength of 530 nm/590 nm and MIC_99_ value (MIC_99_ is the concentration of compound that showed 99% inhibition of bacterial growth) was calculated as described previously (26, 28, 29). Rifampicin was used as a positive control in the assay. Further, the results were confirmed by spotting 5 µL of the culture from each well on MB7H11 agar plates, followed by incubation at 37°C for 3–4 weeks to ascertain that the blue wells did not contain viable bacteria. Moreover, CFU analysis was performed at selected concentrations of inhibitors by plating appropriate serial dilutions of the sample on 7H11 agar plates and incubating the plates at 37°C for 3–4 weeks.

### Checkerboard assay to evaluate drug-drug interaction

The interaction between the compounds B59 and B20 was evaluated by employing a standard 2-D checkerboard assay in a 96-well plate by serially diluting the compounds along the *x*-axis and *y*-axis (34). Briefly, compound B59 was serially diluted along the *x*-axis at a starting concentration of 0.156 µg/mL to 40 µg/mL and compound B20 was serially diluted along the *y*-axis at a concentration range of 2.5 µg/mL to 40 µg/mL. The compounds were then incubated with *M. tb* H37Rv culture at a final A_600nm_ of 0.02 for 7 days at 37°C, and the growth was analyzed by resazurin dye-based method, and MIC_99_ values were calculated by drop plating. MIC_99_ is considered as the concentration of the well that showed no visible growth on the agar plate. Subsequently, the FIC for each compound was calculated by using the formula, FIC = MIC_99_ of compound A in the presence of compound B/MIC_99_ of compound A alone. The ΣFIC was determined as the sum of FIC_A_ and FIC_B_ (ΣFIC value of ≤0.5 represents synergy, ΣFIC value of >4.0 represents antagonism, and no interaction was denoted by ΣFIC value ranging from 0.5 to 4.0) (43).

### Cytotoxicity assays

The cytotoxicity of all the compounds was evaluated by employing REMA assay for various mammalian cell lines such as THP-1 (human monocytic macrophage cell line), HepG2 (hepatocellular carcinoma cell line), and MCF-7 (human adenocarcinoma cell line). Briefly, 1 × 10^4^ cells per well were seeded in a 96-well flat bottom plate in their respective media (RPMI medium for THP-1 and DMEM medium for HepG2 and MCF-7, reconstituted with of 10% FBS and 1% antibiotic-antimycotic mix) in the presence of various concentrations of the compounds ranging from 1 µg/mL to 200 µg/mL. The plates were incubated at 37°C, 5% CO_2_ for 48 hours, followed by the addition of 30 µL of 0.01% resazurin reagent. The fluorescent intensity was measured post 24 hours of incubation at an excitation/emission wavelength of 530 nm/590 nm. The CC_50_ values (CC_50_ value is the concentration of the compound that results in 50% reduction in the fluorescence intensity) were determined for each compound by plotting a graph of fluorescent intensity against the concentrations of compound.

### Intracellular inhibition assays in THP-1 macrophages

The inhibitory potential of compounds against the growth of *M. tb* inside macrophages was evaluated by employing a protocol described previously (24). Briefly, GFP-expressing *M. tb* H37Rv cells were grown till mid-log phase and washed with MB7H9 medium and a single cell suspension was prepared as previously described (24). The CFU/mL of the single-cell bacterial suspension was estimated based on OD_600nm_, and PMA-activated THP-1 cells were infected with *M. tb* H37Rv-GFP at a multiplicity of infection MOI of 1:5 (macrophage/bacteria) in suspension with constant shaking at 100 rpm for 2 hours at 37°C. After infection, the cells were harvested, washed, and resuspended in RPMI media containing 200 µg/mL amikacin for removal of any extracellular bacteria. Following that, cells were counted by Trypan blue exclusion staining and 1 × 10^5^ cells were seeded in a 96-well flat bottom plate containing varying concentrations (1.0 µg/mL–100 µg/mL) of compounds in a final volume of 250 µL.

Alongside, uninfected THP-1 cells were also seeded at the same density in the presence of the same concentration of compounds for assessing the toxicity of compounds toward THP-1 cells. All the plates were kept at 37°C, 5% CO_2_ incubation for 5 days. Subsequently, a preliminary analysis of the *M. tb* growth inhibition was carried out by visualizing the GFP fluorescence under the microscope. THP-1 cells were then lysed by the addition of 100-µL autoclaved double distilled water, and plates were kept at 37°C, 5% CO_2_ incubator 2 hours. Afterwards, the cells were pipetted vigorously and a 50-µL sample was spotted on MB7H11 agar plates and 50 µL was employed for CFU analysis. The MB7H11 agar plates were then incubated at 37°C for 2–3 weeks to visualize the growth of bacteria. MIC_90_ values were determined by plotting a graph of percent inhibition versus concentration of the inhibitor. To the uninfected THP-1 cells, 30 µL of 0.01% resazurin reagent was added and fluorescence was measured at 530 nm/590 nm excitation/emission wavelength the next day.

### Evaluation of the target specificity

To evaluate the target specificity of compound B59 inside mycobacteria, a DapB-overexpressing *M. tb* strain, *M. tb.pFICTO-dapB* (overexpressing a 3x-flag-tagged DapB under tetracycline regulation in addition to the genomic copy) was employed. This overexpressing strain was generated by electroporating a plasmid, *pFICTO-dapB* (chloramphenicol resistant) (pFICTO plasmid was a kind gift from Dr. Vinay Nandicoori, Centre for Cellular and Molecular Biology, Hyderabad) containing the *dapB* gene under a tetracycline promoter in a ‘TET-OFF’ System. The overexpressing strain expresses 3x-flag-tagged DapB in the absence of tetracycline induction. The overexpression of DapB in *M. tb.pFICTO-dapB* was confirmed by immunoblotting studies by using DapB-specific antibody. *Mycobacterium tuberculosis* FadD13, a mycobacterial cytosolic protein unrelated to the DAP pathway, was employed as a loading control. Anti-FadD13 primary antibody was employed for detection of FadD13. Further, MIC_99_ of the compound B59 was determined against *M. tb* H37Rv and *M. tb.pFICTO-DapB* by resazurin dye reduction assay and drop plating on 7H11 agar as described above. Rifampicin and only cells (*M. tb* H37Rv and *M. tb.pFICTO-DapB*) were employed as controls in the study.

## SUPPLEMENTAL MATERIAL

Supplemental figures spectrum.01359-23-s0001.pdf Fig. S1 to S9.

ASM does not own the copyrights to Supplemental Material that may be linked to, or accessed through, an article. The authors have granted ASM a non-exclusive, world-wide license to publish the Supplemental Material files. Please contact the corresponding author directly for reuse.
