# Supplementary material for: In silico virtual screening for the identification of novel inhibitors against dihydrodipicolinate reductase (DapB) of Mycobacterium tuberculosis, a key enzyme of diaminopimelate pathway
Source: Microbiol Spectr. 2023 Oct 19;11(6):e01359-23. doi: 10.1128/spectrum.01359-23 (PMC10714930; doi:10.1128/spectrum.01359-23)
Supplement: Supplemental figures — Fig. S1 to S9. [file spectrum.01359-23-s0001.pdf]

SUPPLEMENTARY FIGURES

Figure S1

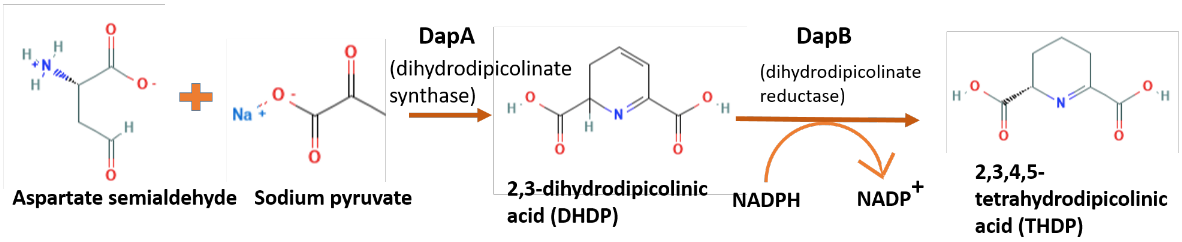

FIG S1 Reaction catalyzed by dihydrodipicolinate reductase (DapB). The substrate of DapB, 2,3- dihydrodipicolinate (DHDP) is formed through aldol condensation between pyruvate and ASA in a reaction catalysed by dihydrodipicolinate synthase (DapA). Further, 2,3-dihydrodipicolinate (DHDP) is reduced to 2,3,4,5-tetrahydrodipicolinic acid (THDP) in a reaction catalyzed by DapB by using NADPH as a cofactor.

Figure S2

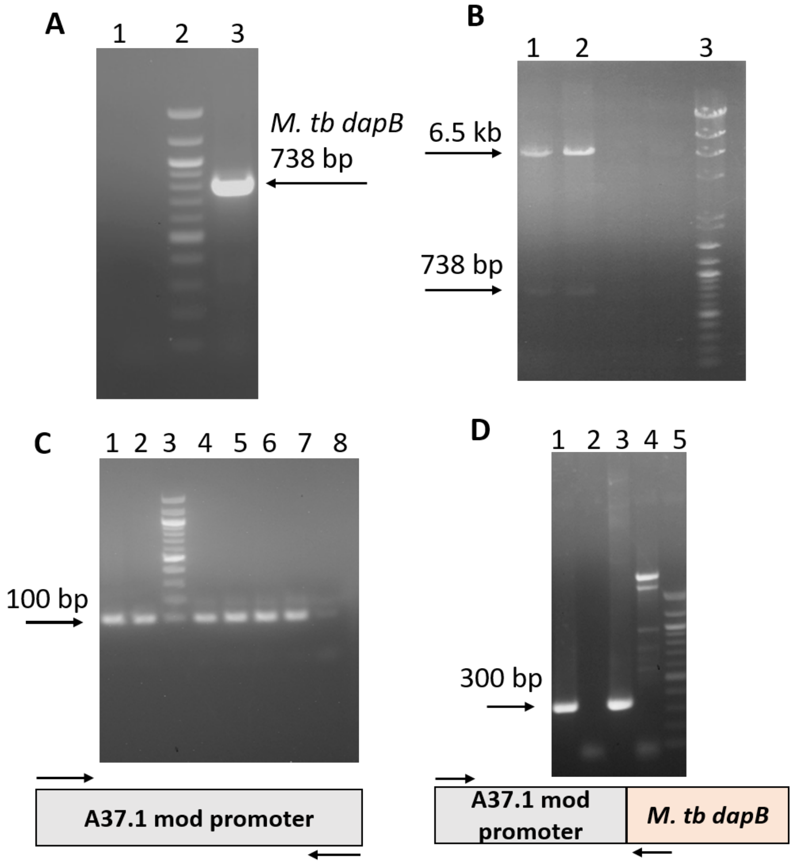

FIG S2 Characterisation of *M. tb dapB* knockdown mutant strain. (A) PCR amplification of dapB gene (~700 bp) using *M. tb* H37Rv genomic DNA (lane 3). Lane 1- no template control; lane 2- 100 bp marker. (B) Screening of the recombinant *E. coli* colonies for the presence of dapB antisense plasmid by restriction digestion (lane 1 and 2); Lane 3- λhindIII DNA ladder and 100 bp marker. (C) Screening of colonies obtained after electroporation of *dapB* antisense knockdown mutant construct and vector control construct by PCR using primers for the amplification of A37.1mod promoter (100 bp). Lane 1- *M. tb* pSD5A37.1mod/dapB-AS; lane 2- positive control (pSD5A37.1mod/dapB-AS plasmid); lane 3- 100 bp marker; lane 4-6 - *M. tb* pSD5A37.1mod, lane 7- positive vector control (pSD5A37.1mod plasmid) and lane 8- no template control (D) Screening of colonies obtained after electroporation of antisense construct *(M. tb* pSD5A37.1 mod/dapB-AS) by PCR using primers (A37.1 mod as a forward primer and *dapB* specific reverse primer). Lane 1- plasmid DNA from *M. tb* antisense knockdown mutant strain; lane 2- no template control; lane 3- plasmid DNA employed for electroporation; lane 4- *M. tb* genomic DNA and lane 5- 100 bp marker.

Figure S3

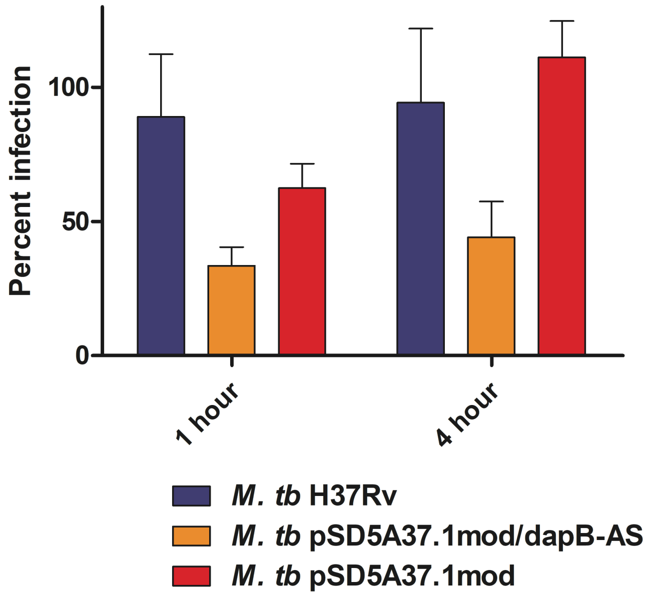

FIG S3 Confocal microscopy studies for measuring the rate of intracellular infection of *M. tb* strains. THP-1 macrophages were infected with FITC labelled *M. tb* strains at MOI of 1:5 followed by visualisation under confocal microscope at 1 hour and 4 hour post infection. Bar graph represents percent infection of macrophages with different FITC labelled *M. tb* strains.

Figure S4

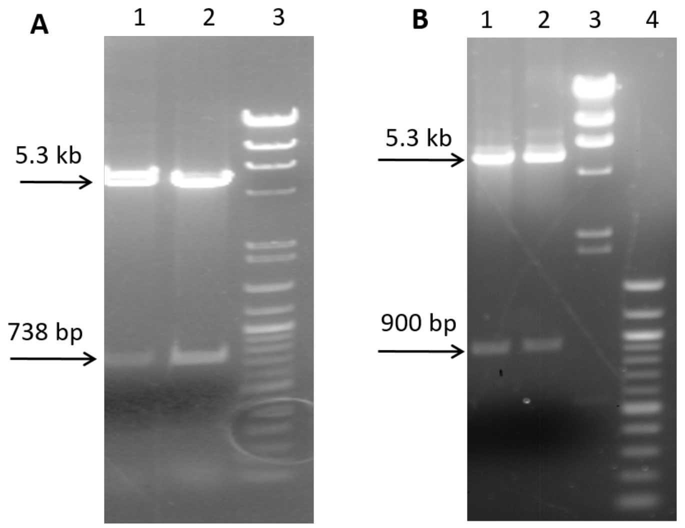

FIG S4 Characterization of recombinant *E. coli* clones by restriction digestion. (**A)** Restriction digestion was performed with NdeI and XhoI to screen *E. coli* clones containing recombinant construct, pET28a/DapB, resulting in a fall out of approximately 738 bp in both clone 1 (lane 1) and clone 2 (lane 2). Lane 3 denotes λhindIII DNA ladder and 100 bp marker. **(B)** Restriction digestion was performed with HindIII and XhoI to screen *E. coli* clones carrying recombinant pET28a/DapA plasmid, resulting in a fall out of approximately 900 bp in clone 1 (lane 1) and clone 2 (lane 2). Lane 3 and lane 4 denotes λhindIII DNA ladder and 100 bp marker, respectively.

Figure S5

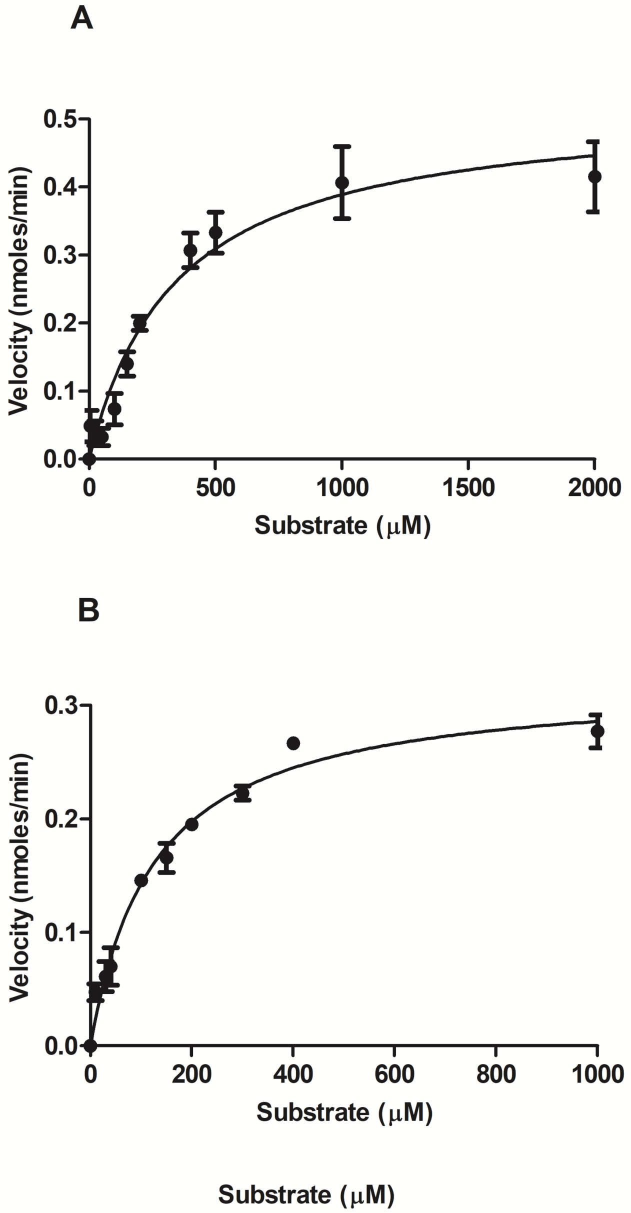

FIG S5 Determination of K_m values for the substrates ASA (A) and sodium pyruvate (B). The enzymatic activity was determined at varying concentrations of the substrates by using the coupled enzyme assay. The K_m was determined with nonlinear regression analysis using Michaelis –Menten plot in Graphpad prism. The data is represented as mean ± SEM from at least two independent experiments.

Figure S6

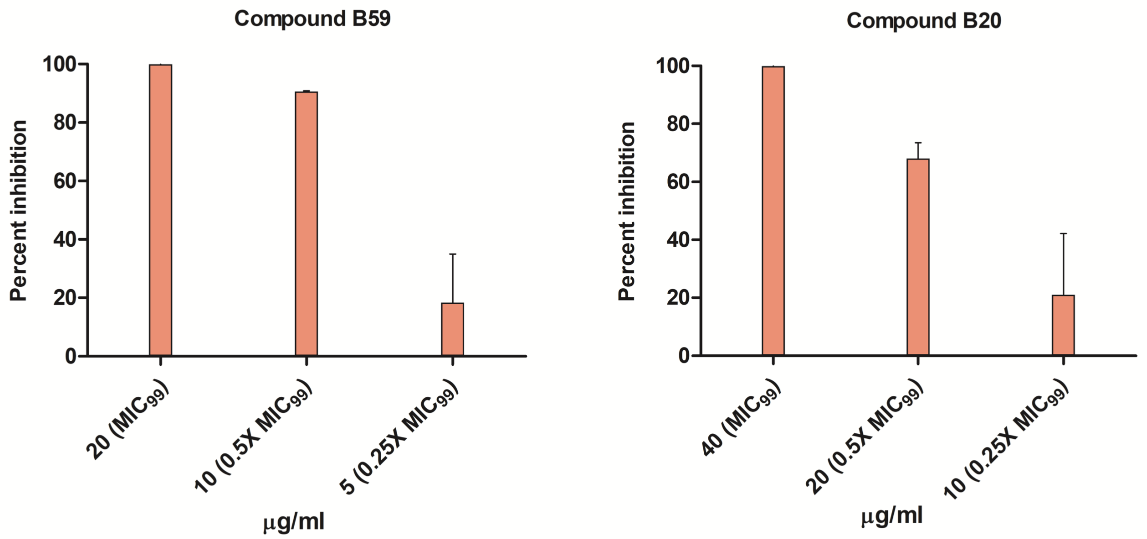

FIG S6 Evaluation of the effect of varying concentrations of compounds, B20 and B59 on the growth of *M. tb.* The bacterial cells were incubated with varying concentrations of compounds and CFU enumeration was carried out for the wells having concentrations at MIC_{99}, 0.5X MIC_{99}, 0.25X MIC_{99}.

Figure S7

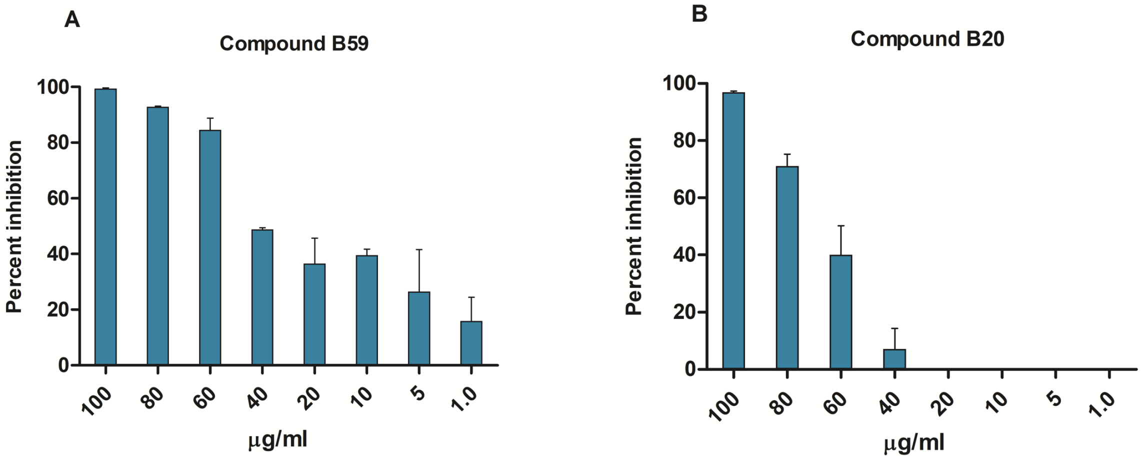

FIG S7 Dose response inhibition curve for compounds B59 and B20 to determine their intracellular MIC_{90} values. PMA activated THP-1 macrophages were infected with *M. tb* H37Rv in the presence of varying concentrations of compounds, B59 and B20 and the growth of the pathogen was evaluated by CFU enumeration on agar plates after 5 days of infection and percent inhibition was calculated. The data represents mean ± SEM of two independent experiments.

Figure S8

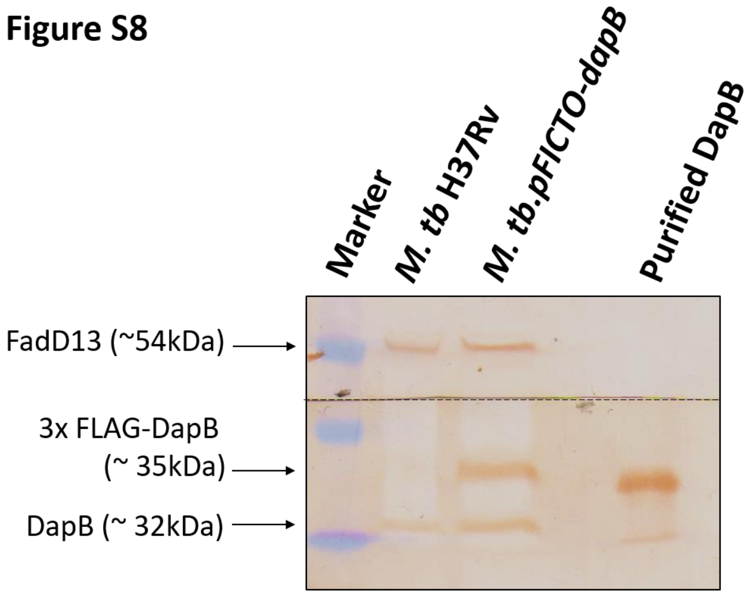

FIG S8 Expression analysis of DapB in wild type *M. tb H37Rv* and *M. tb.pFICTO-dapB* strains. 20 μg of protein lysates of *M. tb H37Rv* and *M. tb.pFICTO-dapB* were electrophoresed on 12.5% polyacrylamide gel. The proteins were transferred on PVDF membrane and the blot was cut into two at the dashed line. Immunoblotting was carried out by using anti-DapB for the lower part of the membrane and with anti-fadD13 polyclonal antibody for the upper part. (Marker size- 50, 37 and 25 kDa from top to bottom).

Figure S9

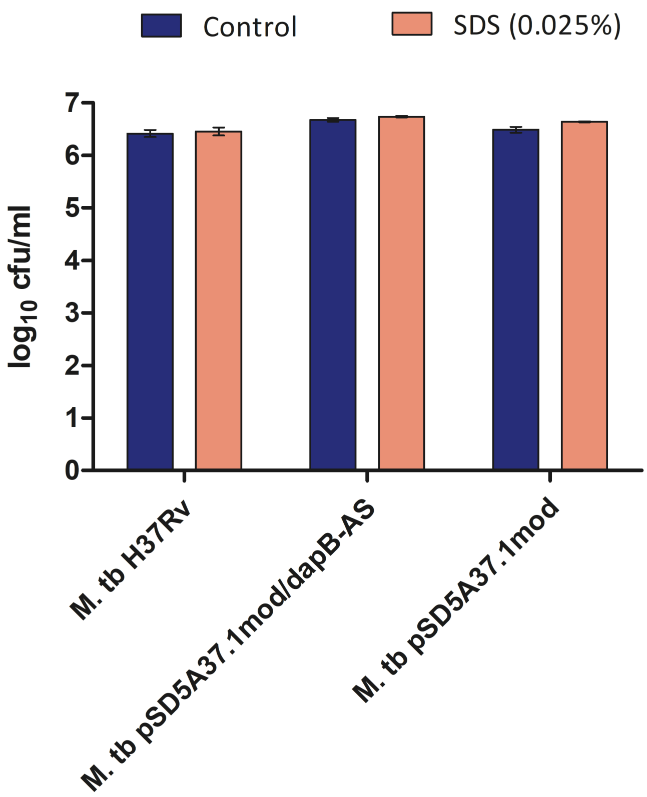

FIG S9 Evaluation of the effect of 0.025% SDS on the extracellular growth of different *M. tb* strains. Bar graph represents $\log_{10}$ CFU/ml values of different strains after treatment with 0.025% SDS for 10 minutes.
